# Rediscovery of *Osteocephalus vilarsi* (Anura: Hylidae): an overlooked but widespread Amazonian spiny-backed treefrog

**DOI:** 10.7717/peerj.8160

**Published:** 2019-12-04

**Authors:** Miquéias Ferrão, Jiří Moravec, Leandro J.C.L. Moraes, Vinicius T. de Carvalho, Marcelo Gordo, Albertina P. Lima

**Affiliations:** 1 Museum of Comparative Zoology, Harvard University, Cambridge, MA, USA; 2 Programa de Pós-Graduação em Ecologia, Instituto Nacional de Pesquisas da Amazônia, Manaus, Amazonas, Brazil; 3 Department of Zoology, National Museum, Prague, Czech Republic; 4 Programa de Capacitação Institucional, Instituto Nacional de Pesquisas da Amazônia, Manaus, Amazonas, Brazil; 5 Departamento de Biologia, Universidade do Amazonas, Manaus, Amazonas, Brazil; 6 Coordenação de Biodiversidade, Instituto Nacional de Pesquisas da Amazônia, Manaus, Amazonas, Brazil

**Keywords:** Amphibia, Amazonia, Brazilian biodiversity, Integrative taxonomy, Advertisement call, Tadpoles, White-sand ecosystems

## Abstract

*Osteocephalus vilarsi* (Melin, 1941) is an Amazonian treefrog species known for over 75 years from its holotype only. Due to a lack of published data on its morphological diagnostic characters and their variations, as well as the absence of molecular, acoustic and ecological data supporting its identity, a highly dynamic taxonomic history has led this species to be confused and even synonymised with other *Osteocephalus* species from distinct species groups. The molecular phylogenetic relationships of *O. vilarsi* were investigated based on recently collected specimens from eight Northwestern Brazilian localities in the state of Amazonas, leading to its removal from the *Osteocephalus taurinus* species group and placement in the *Osteocephalus planiceps* species group. Furthermore, detailed data on morphology and colour variation are provided, as well as advertisement call and tadpole descriptions. Finally, the currently known geographic range of *O. vilarsi* is considerably extended, first data on the natural history of the species are provided, and the possible ecological preference of *O. vilarsi* for Amazonian white-sand forests is discussed.

## Introduction

The spiny-backed treefrogs belonging to the genus *Osteocephalus* Steindachner, 1862 are medium to large-sized arboreal frogs inhabiting primary and secondary forests in the vast area of the Orinoco and Amazonian basins (Frost, 2019). They are distributed from Colombia, Venezuela and the Guyanas to Bolivia and Brazil (Jungfer et al., 2013) and their altitudinal range varies from sea level in Venezuela, the Guianas, Brazil (e.g. *O. taurinus* Steindachner, 1862) to up to 2,200 m in Ecuador (e.g. *O. festae* Peracca, 1904). With 24 currently recognised species, the genus *Osteocephalus* represents the most specious genus of the subfamily Lophyohylinae Miranda-Ribeiro, 1926 (Frost, 2019). The increasing attention paid to spiny-backed treefrog systematics and biogeography in the last decade (Moravec et al., 2009; Jungfer, 2010; Ron et al., 2010, 2012; Hoogmoed, 2013; Jungfer et al., 2013, 2016; Moravec et al., 2016) has significantly improved our knowledge on *Osteocephalus* phylogeny, resulting in nomenclatural and biogeographical corrections, descriptions of new taxa and the definition of new candidate species. Among the presently accepted species, *O. vilarsi* (Melin, 1941) is known only from its holotype and represents the most enigmatic species of *Osteocephalus* (Jungfer et al., 2013; Frost, 2019).

*Osteocephalus vilarsi* was originally described as *Hyla* (*Trachycephalus*) *vilarsi*, based on a gravid female collected by indigenous people at Missão Taracuá, in 1924. This mission is located on the right bank of the upper Uaupés River (in the state of Amazonas, Brazil, 00°07′56′N, 68°33′03″W, ca 100 m a.s.l.), a tributary of the Negro River (Caldwell, Lima & Keller, 2002). Melin (1941) highlighted the similarity between *O. vilarsi* and *O. taurinus*, but, nevertheless, differentiated the new species only from *O. planiceps* Cope (1874). *O. vilarsi* has experienced taxonomic problems since its description. First, the species was synonymised with *O. taurinus* by Bokermann (1966), without further justification. A few years later, Cochran & Goin (1970) directly compared two specimens of *O. leprieurii* (Duméril & Bibron, 1841; MCZ 28042, CNHM 69716) from Colombia to the holotype of *O. vilarsi* and considered the latter name a junior synonym of *O. leprieurii*. Trueb & Duellman (1971) disagreed with the allocation of *O. vilarsi* in the synonymy of *O. leprieurii* and argued that *O. vilarsi* was a junior synonym of *O. taurinus* based on the fact that both species share moderately exostosed dermal roofing bones, distinctly elevated lateral frontoparietal edges and spots on the throat, chest and flanks. *O. vilarsi* was considered the junior synonym of *O. taurinus* for 30 years, until its resurrection as a valid species by Jungfer (2010). In his study, Jungfer redescribed in detail the holotype of *O. vilarsi* and compared it to other *Osteocephalus* species, mainly *O. taurinus*, *O. planiceps* and *O. leprieurii*. In addition, Jungfer (2010) highlighted that specimens reported as *O. planiceps* by Gordo & Neckel-Oliveira (2004) from the Jaú National Park on the lower Negro River (the only Brazilian record for *O. planiceps*) may represent *O. vilarsi* instead of *O. planiceps*.

In a taxonomic and systematic revision based on molecular and morphological data, Jungfer et al. (2013) proposed and defined five *Osteocephalus* species groups. Due to external morphology similarities (genetic data were not available for *O. vilarsi*) these authors tentatively associated *O. vilarsi* to the *O. taurinus* specie*s* group. In the same study, Jungfer et al. (2013) revealed nine candidate species for the *Osteocephalus* genus, including *O. planiceps* (Ca1_Neblina411). This candidate species is known from one specimen collected in Venezuela, close to the Brazilian border (Neblina Base Camp, Rio Mawarinuma, Amazonas; ca 140 m a.s.l.). Neblina Base Camp is located ca. 275 km east of Missão Taracuá, the type locality of *O. vilarsi*. The vegetation of both localities is characterised as a white-sand forest (Adeney et al., 2016) and both the holotype of *O. vilarsi* and the specimen of *O. planiceps* (Ca1_Neblina411) (sensu Jungfer et al., 2013) were collected at a similar elevation. With regard to this, it is interesting that specimens of *O. planiceps* reported from Jaú National Park (Gordo & Neckel-Oliveira, 2004), supposed to be *O. vilarsi* (Jungfer, 2010), were also collected in a white-sand forest.

In the frame of herpetological surveys of white-sand forest areas in the vicinity of the municipality of Novo Airão (west bank of Negro River, in the state of Amazonas, Brazil) carried out from 2015 to 2018, adults, juveniles and tadpoles belonging to a species of *Osteocephalus* morphologically corresponding to the description of *O. vilarsi* and genetically clustering with *O. planiceps* CA1 (sensu Jungfer et al., 2013) were collected. After morphological comparison of these specimens to the holotypes *of O. planiceps* and *O. vilarsi*, as well as to museum specimens of other *Osteocephalus* species, we attributed these individuals to *O. vilarsi*. Subsequently, we confirmed our determination by comparison of mitochondrial DNA sequences from these specimens with the DNA of individuals collected directly at the type locality of *O. vilarsi*.

**Figure 1:**
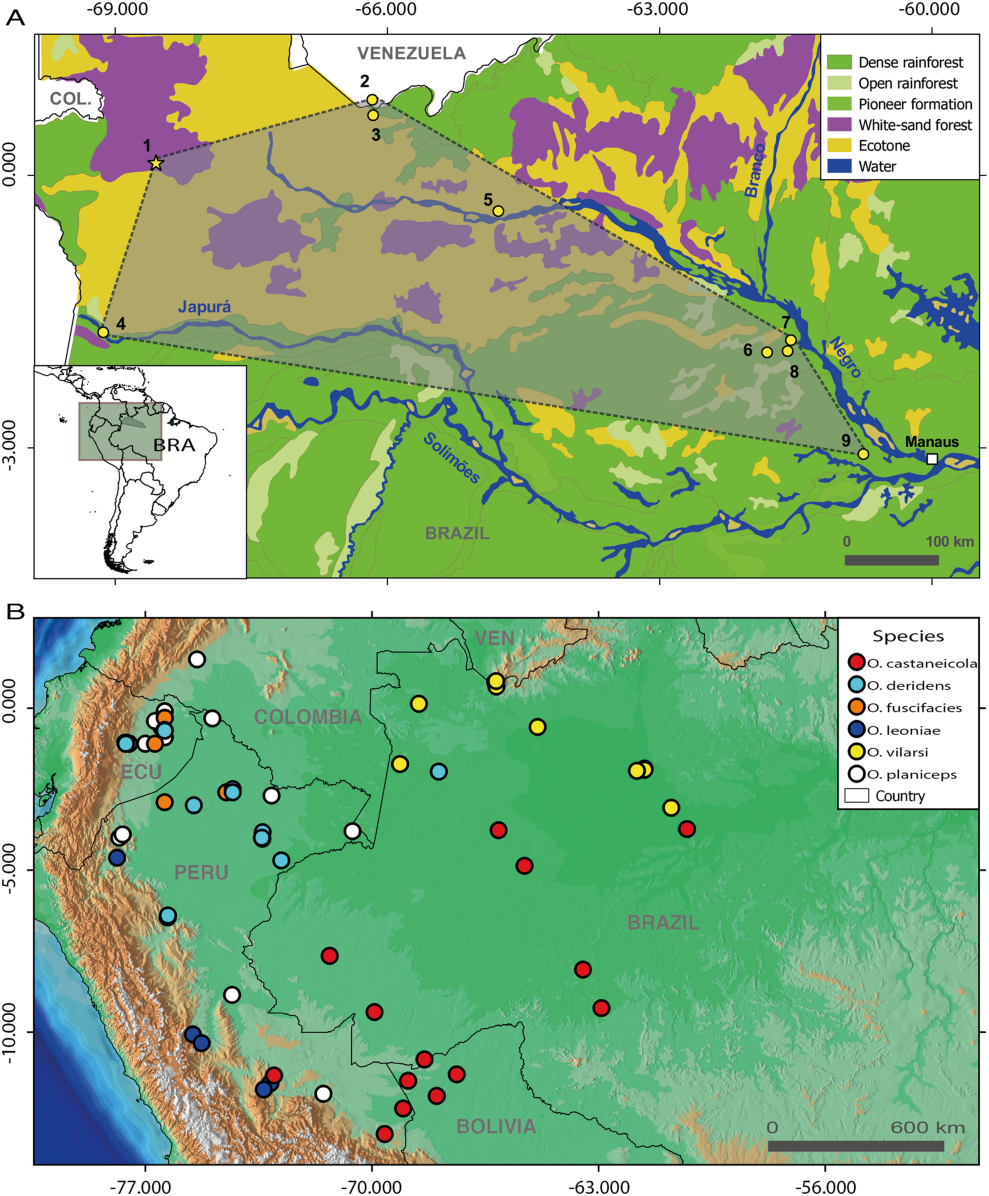
Geographic distribution of members of the *Osteocephalus planiceps* species group. (A) Distribution of *O. vilarsi* in north-western Brazil and southern Venezuela on a vegetation background map. (B) Distribution of all species of *O. planiceps* species group on an altitudinal background map according to Jungfer et al. (2013), GBIF (www.gbif.org) and own unpublished records. Localities in map A (all in the state of Amazonas, Brazil, except for locality 2 in the Amazonas Department, Venezuela): 1, Missão Taracuá; 2, Venezuelan slope of Pico da Neblina; 3, Brazilian foothills of Pico da Neblina; 4, left bank of Japurá River; 5, Ayuanã River, Santa Isabel do Rio Negro; 6, Miratucu Lake, Jaú National Park; 7, Seringalzinho Village, Jaú National Park; 8, Jaú National Park; 9, Rio Negro Sustainable development reserve. A star denotes the type locality of *O. vilarsi* (Missão Taracuá). Vegetation types according to Instituto Brasileiro de Geografia e Estatística (1992).

Further revision of available *Osteocephalus* collections revealed that the specimens of *Osteocephalus* reported from Jaú National Park by Gordo & Neckel-Oliveira (2004) actually represent *O. vilarsi*, and that additional voucher specimens of this species from the Park territory are available. Moreover, specimens have already been sampled at several other localities in the west and north regions of the Negro River (Amazonas, Brazil; see Material and Methods and Fig. 1). Recently, the name *O. vilarsi* was used for specimens collected in Santa Isabel do Rio Negro area (Amazonas, Brazil) by Menin et al. (2017), but without providing any data on the morphology of these frogs or any explanation concerning the determination method. Examination of the respective voucher specimens (see Appendix 1 in Menin et al., 2017) revealed that all seven specimens listed as *O. vilarsi* represent a species belonging to the *O. taurinus* group. Interestingly, only one actual specimen of *O. vilarsi* (CZPB-AA 239) from Santa Isabel do Rio Negro material exists, but it was wrongly determined as *O. planiceps* (in addition, the second specimen listed by the above authors as *O. planiceps*, CZPB-AA 240, was determined incorrectly and represents a species belonging to the *O. taurinus* group).

In this study, we provide a detailed phylogeny of *Osteocephalus* and report detailed data on morphological variation and colouration of *O. vilarsi*, also describing its advertisement call and tadpoles. Finally, we extend the known range of *O. vilarsi*, provide first data on its natural history, and discuss the possible association of this species with Amazonian white-sand forests.

## Materials and Methods

### Examined material

The examined material attributed to *O. vilarsi* consists of 29 adults, two juvenile specimens and 10 tadpoles collected by different researchers from eight Brazilian localities in the state of Amazonas (for details see Fig. 1A; Table S1). This material includes three adult females from the type locality of *O. vilarsi* (Missão Taracuá) (Appendix I). In addition, we analysed DNA sequences of the only known Venezuelan specimen (AMNH 131254) collected in the Department of Amazonas close to the Brazilian border (Fig. 1A; Table S2). This individual was first reported as *O. leprieurii* by Faivovich et al. (2004) and, later, as new candidate species *O. planiceps* (Ca1_Neblina411) by Jungfer et al. (2013).

Specimens of *O. vilarsi* collected by us in the Brazilian localities Novo Airão and São Gabriel da Cachoeira were recorded at night through visual encounters. Tadpoles were collected in a temporary shallow pond on white-sand soil at Rio Negro Sustainable Development Reserve (RDS Rio Negro). Adults were anesthetised and killed with a topic 2% Benzocaine solution, fixed in 10% formalin and stored in 70% alcohol. Tadpoles were killed with a 5% lidocaine solution and preserved in 5% formalin. Before fixation of recently collected specimens (RDS Rio Negro and Pico da Neblina), tissue samples from adults and one tadpole were extracted, conserved in pure alcohol and stored at the Laboratory of Albertina Lima and Collection of Genetic Resources at Instituto Nacional de Pesquisas da Amazônia (INPA), Manaus, Brazil. Adults and tadpoles were deposited at INPA’s zoological collection herpetological section (INPA-H). See Menin et al. (2017) for sampling methods in the municipality of Santa Isabel do Rio Negro.

Specimens were collected under ICMBio/RAN permit (Reg. 659755 Nos. 13777 and 52206-1). ICMBio and RAN are institutions of the Ministry of Environment, Government of Brazil. These permits were subject to approval of all procedures for collecting and euthanizing frogs.

Collections acronyms are as follows: AMNH, American Museum of Natural History, New York, USA; APL, Albertina P. Lima field numbers, INPA, Manaus, Brazil; AJC, Andrew J. Crawford field numbers; CBF, Colección Boliviana de Fauna, La Paz, Bolivia; CFBH, Célio F.B. Haddad field numbers, UNESP, Rio Claro, Brazil; CORBIDI, Centro de Ornitología y Biodiversidad, Lima, Peru; CZPB-AA, herpetological section of the Coleção Zoológica Paulo Bührnhein, Manaus, Brazil; EPN, Escuela Politecnica Nacional, Quito, Ecuador; GGU, Giussepe Gagliardi-Urrutia field numbers at UNAP, Iquitos, Peru; GNM, Göteborg Natural History Museum; INPA-H, herpetological section of the zoological collection of INPA; IRSNB, Royal Belgian Institute of Natural Sciences, Brussels, Belgium; KHJ-F, Karl-Heinz Jungfer field numbers; MAR, Marco Rada field numbers; MCZ-A, Harvard Museum of Comparative Zoology, Cambridge, USA; MG, Marcelo Gordo field number; MHNLS, Museo de Historia Natural La Salle, Caracas, Venezuela; MHNC, Museo de Historia Natural, Universidad Nacional de San Antonio Abad del Cusco, Cusco, Peru; MUSM, Museo de Historia Natural de la Universidad de San Marcos, Lima, Peru; MSH, Marinus S. Hoogmoed field numbers; MTR, Miguel T. Rodrigues field numbers; MZUSP, Museu de Zoologia da Universidade de São Paulo, São Paulo, Brazil; NMP-P6V, National Museum, Prague, Czech Republic; QCAZ, Museo de Zoología, Pontifica Universidad Católica del Ecuador, Quito, Ecuador; SMNS, Staatliches Museum für Naturkunde, Stuttgart, Germany; SMS, Sergio Marques de Souza field numbers; TG, Taran Grant field numbers; UA, Universidad de los Andes, Bogotá, Colombia.

### Species identification

The collected specimens were attributed to *O. vilarsi* on the basis of morphological comparison with the female holotype of *O. vilarsi* (NHMG 488) through photographs and data presented in its redescription available in Jungfer (2010). Apart from that, only two species of *Osteocephalus* occurring in Missão Taracuá (the type locality of *O. vilarsi*) possess frontoparietal ridges on the head: *O. vilarsi* and the morphologically clearly different *O. taurinus*. In our phylogeny, individuals of *O. vilarsi* from Missão Taracuá clustered with all individuals attributed to *O. vilarsi* from other localities. The intraspecific genetic distance among them was very short even between specimens from geographically distant localities.

### DNA extraction and phylogenetic analyses

Genomic DNA was extracted from 11 samples of *O. vilarsi*: three adults from the type locality (Missão Taracuá), three adults and one juvenile from the Brazilian foothills of the Neblina mountain range, three adults and one tadpole from RDS Rio Negro. Additionally, we extracted genomic DNA from one specimen of *O. taurinus* from Missão Taracuá and one tadpole of *O. taurinus* from northern Purus-Madeira Interfluve (Amazonas, Brazil). Extractions were obtained through the Wizard Genomic DNA Purification kit (Promega, Madison, WI, USA), following manufacturer protocols. Primers 16Sar and 16Sbr (Palumbi et al., 1991) were used to amplify a ~553 (476–588) bp-long fragment of the 16S rRNA mitochondrial gene. Polymerase chain reaction (PCR) protocols included a reaction mix with final volume of 15 μL, containing 2.0 μL ddH2O, 1.5 μL 25 mM MgCl2, 1.5 μL 10 mM dNTPs (2.5 mM of each dNTP), 3 μL 5X amplification buffer (75 mM Tris HCl, 50 mM KCl, 20 mM (NH4) 2SO4), 1.5 μL 2 μM solution of each primer, 0.3 μL Taq DNA polymerase 5 U/μL (Biotools, Madrid, Spain) and 1.5 μL of genomic DNA (about 30 ng/μL). Fragment amplification involved a pre-heating step at 73 °C for 60 s, followed by 35 denaturation cycles at 94 °C for 10 s, primer annealing at 50 °C for 35 s and an extension at 72 °C for 90 s, followed by a final extension step at 72 °C for 10 min. Sequencing reactions were carried out after PCR purification using exonuclease and thermosensitive alkaline phosphatase, following the manufacturer’s recommendations (Thermo Fisher Scientific, Waltham, MA, USA) and followed by the use of aABI BigDye Terminator Cycle Sequencing Kit following the manufacturer’s instructions. Forward and reverse primers were used in the sequencing reactions (annealing temperature of 50 °C) and resolved on an ABI 3130xl automatic sequencer. Sequences were manually verified at Geneious (Kearse et al., 2012).

In order to infer the phylogenetic relationships of *O. vilarsi*, we selected 80 sequences of 16S, 81 sequences of 12S rRNA (12S), 43 sequences of NADH dehydrogenase subunit 1 (ND1), 30 sequences of cytochrome oxidase I (COI) and 27 sequences of cytochrome b (CYTB) from GenBank corresponding to 81 specimens of *Osteocephalus* and four specimens of *Dryaderces*. The final matrix was composed by 98 terminals representing all nominal species of *Osteceophalus* (except *O. duellmani*) and two candidate species revealed by Jungfer et al. (2013) plus two specimens of *Dryaderces pearsoni* (Gaige, 1929) and two specimens of *Dryaderces* sp. CA1 used as outgroups (Table S2). Nuclear markers were not included in the dataset due to the absence of available sequences for approximately 90% of the analysed samples.

Sequences of each marker were aligned separately in Bioedit (Hall, 1999) using the ClustalW algorithm (Thompson, Higgins & Gibson, 1994) and manually checked. Alignments were concatenated in MESQUITE 3.5 (Maddison & Maddison, 2018) and the final matrix was constituted of 4382 bp. PartitionFinder 2.1.1 (Lanfear et al., 2017) was used to infer the best nucleotide evolution model and partitions schemes through PhyML (Guindon et al., 2010) and Bayesian Information Criterion. The best-fit partition scheme and model evolution are displayed in Table 1.

**Table 1:** Best-fit partition schemes and nucleotide evolution models determined by PartitionFinder.

Schemes	Best model	Subset partitions	Subset alignment
1	GTR+I+G	12S, 16S, ND1\1, CYTB\2	1–950, 951–2383, 3037–3997, 3999–4382
2	K80+I+G	COI\1	2384–3036
3	HKY+I	COI\2, ND1\2, CYTB\3	2385–3036, 3038–3997, 4000–4382
4	GTR+I+G	COI\3, ND1\3, CYTB\1	2386–3036, 3039–3997, 3998–4382

**Note:**

Numbers after slashes represent codons of protein coding markers.

The phylogenetic relationship was reconstructed using Bayesian Inference (BI). The BI tree was inferred in MrBayes 3.2.6 (Ronquist et al., 2012) using four runs of 10 million generations with a Metropolis-coupled Markov chain Monte Carlo algorithm. Each run comprised four Markov chains, with probabilities sampled every 1,000 generations. Convergence among runs and model stationarity parameters were verified using Tracer 1.7 (Rambaut et al., 2018). Tree files were combined in LogCombiner 1.8.4 (Drummond et al., 2012) after discarding 25% of the trees. The majority rule consensus tree was built using TreeAnnotator 1.8.4 (Drummond et al., 2012). The average Kimura-2-parameter (K2P) (Kimura, 1980) and uncorrected pairwise genetic distances between *O. vilarsi* and the remaining dataset of *Osteocephalus* were estimated using MEGA 6.0 (Tamura et al., 2013).

### Adult morphology

Sex and maturity were inferred through the presence or absence of secondary sexual characters (e.g. vocal sac, vocal slits, skin texture, nuptial excrescences on prepollex, presence of eggs). Measurements are given in mm and were taken to the nearest 0.1 mm using a dissecting microscope and a digital calliper. Nine measurements were taken according to Duellman (1970) as follows: snout–vent length (SVL), head length (HL: distance from the posterior edge of the jaw articulation to the tip of the snout), head width (HW at jaw angle), horizontal eye diameter (ED), tibia length (TL), horizontal tympanum diameter (TD), minimal interorbital distance (IOD), upper eyelid width (UEW), eye-nostril distance (EN). One measurement was taken following Heyer et al. (1990): thigh length (THL). In addition, disc width on Finger III (3FD) and Toe IV (4TD) were also measured.

Some *Osteocephalus* descriptions and redescriptions (Ron & Pramuk, 1999; Jungfer & Lehr, 2001; Jungfer, 2010; Jungfer et al., 2016) have stated that foot length followed Duellman (1970), who stated FL as ‘the distance from the proximal edge of the inner metatarsal tubercle (the large tubercle at the base of the first toe) to the tip of the longest (fourth) toe (including the disc)’. However, the FL is always longer than TL in those studies, which is not consistent with Duellman’s method. In our study, FL was taken from the tip of Toe IV to the heel, in order to make this measurement comparable to those provided in recent *Osteocephalus* studies. Webbing formulae follow the standards of Savage & Heyer (1967) as modified by Myers & Duellman (1982). Colour in life was described based on field notes and on digital photographs.

### Morphological statistics

Although *O. vilarsi* and *O. planiceps* are not sister species according our phylogenetic results and the phylogeny published by Jungfer et al. (2013), these species are morphologically similar. Due to that, we performed a Principal Component Analyses (PCA) with morphometric data in order to investigate if the morphometric multivariate space occupied by these two species overlap. The PCA was performed with 12 morphometric ratios (HL/SVL, HW/SVL, IOD/SVL, EN/SVL, ED/SVL, UEW/SVL, TD/SVL, 3FD/SVL, 4TD/SVL, THL/SVL, TL/SVL, FL/SVL). As the SVL seems an important morphological character to differentiate species of *Osteocephalus*, we also included the SVL in the PCA. Analyses were conducted separately for adult males and females to avoid biases attributed to sexual dimorphism. PCAs were implemented in R environment (R Core Team, 2018) through the function ‘prcomp’ of the package *stats*. We set the parameters ‘scale’. and ‘center’ as ‘TRUE’ to scale and centre morphometric variables, respectively. The numbers of retrieved Principal Components (PCs) were determined through Broken Stick Model. PCA graphs were obtained trough the function ‘autoplot’ of the package *ggplot2* (Wickham, 2016). Adult specimens of *O. planiceps* from Peru and Ecuador housed in the MCZ-A and NMP-P6V collections were used in the PCA and measured as described for *O. vilarsi*. Morphometric measurements of both species are available in Table S3. Results are presented in the subsection Comparaisons.

### Tadpoles

The developmental stages of 10 tadpoles were determined according to Gosner (1960). Eight morphometric measurements were taken according to Altig & McDiarmid (1999) as follow: total length (TL); body length (BL); tail length (TAL); maximum tail height (MTH); tail muscle height (TMH); tail muscle width (TMW); internarial distance (IND); interorbital distance (IOD). Three measurements were taken following Randrianiaina et al. (2011): maximum body width (BW); maximum oral disc width (ODW) and size of the dorsal gap of marginal papillae (DG). Morphometric measurements were taken to the nearest 0.1 mm with a micrometer coupled to a dissecting microscope. Tadpole descriptions follow Schulze, Jansen & Köhler (2015) and were based on five tadpoles in Gosner stage 36. Colour was described based on one tadpole raised in laboratory until metamorphosis.

### Bioacoustics

Advertisement calls of *O. vilarsi* were recorded at two localities in the state of Amazonas, Brazil: (1) RDS Rio Negro, located in the municipality of Novo Airão: calls of one male were recorded using an Olympus LS-14 Linear PCM Recorder on 24 and 25 October 2017 (air temperature 25–26 °C); (2) Seringalzinho Village, Jaú National Park, located in the municipality of Novo Airão: calls of two males were recorded with a Sennheiser ME66 external microphone in combination with a Marantz PMD 670 recorder on 25 April 2000. Air temperature at the moment of recording was not determined. Both recordings were made at a sampling rate of 44.1 kHz, sample size of 16 bits and stored in wave format. Calls of *O. vilarsi* were housed at the SAPOTECA, an advertisement call repository of the Research Programme on Biodiversity (PPBIO/INPA), Manaus, Brazil. Calls can be requested at https://ppbio.inpa.gov.br/en/sapoteca/contact.

Temporal and spectral parameters of 23 calls were measured, seven from the male recorded at RDS Rio Negro (not collected) and 16 of the two males recorded at Seringalzinho Village (INPA-H 40455 = 11 calls; INPA-H 40467 = five calls). Calls were analysed using Raven 1.5 (Bioacoustics Research Programme 2015) through a Blackman window, 3 dB Filter Bandwidth of 80 Hz, overlap of 80%, hop size of 4.1 ms, and DFT size of 2,048 samples. Oscillograms were used to measure the call, note and pulse duration and the inter-note and inter-pulse interval. The number of notes, pulses per notes and harmonics were counted through spectrogram visualisations. The dominant frequency of pulses in each note was determined from power spectrum graphs through the function Peak Frequency. The Seewave package version 2.0.5 (Sueur, Aubin & Simonis, 2008) was used to generate advertisement call figures. Seewave was set up as follows: Hanning window and 512 resolution points (FFT). The note-centered approach (Köhler et al., 2017) was used to describe the advertisement call. Call terminology follows Köhler et al. (2017).

Although the advertisement call of *O. planiceps* has been described by Ron & Pramuk (1999), we analysed 19 advertisement call from three males of this species from Ecuador in order to obtain the same acoustic parameters measured for *O. vilarsi* in the present study. Advertisment calls of *O. planiceps* were downloaded from Anfibios del Ecuador (https://bioweb.bio/faunaweb/amphibiaweb/: Ron, Merino-Viteri & Ortiz, 2019). Recordings were made by Morley Read in: (1) Reserva Biológica Jatun Sacha, Provincia Napo; (2) Parque Nacional Yasuní and (3) Pompeya Sur, cerca del río Napo, both in Provincia Orellana.

## Results

### Molecular phylogenetic analyses

**Figure 2:**
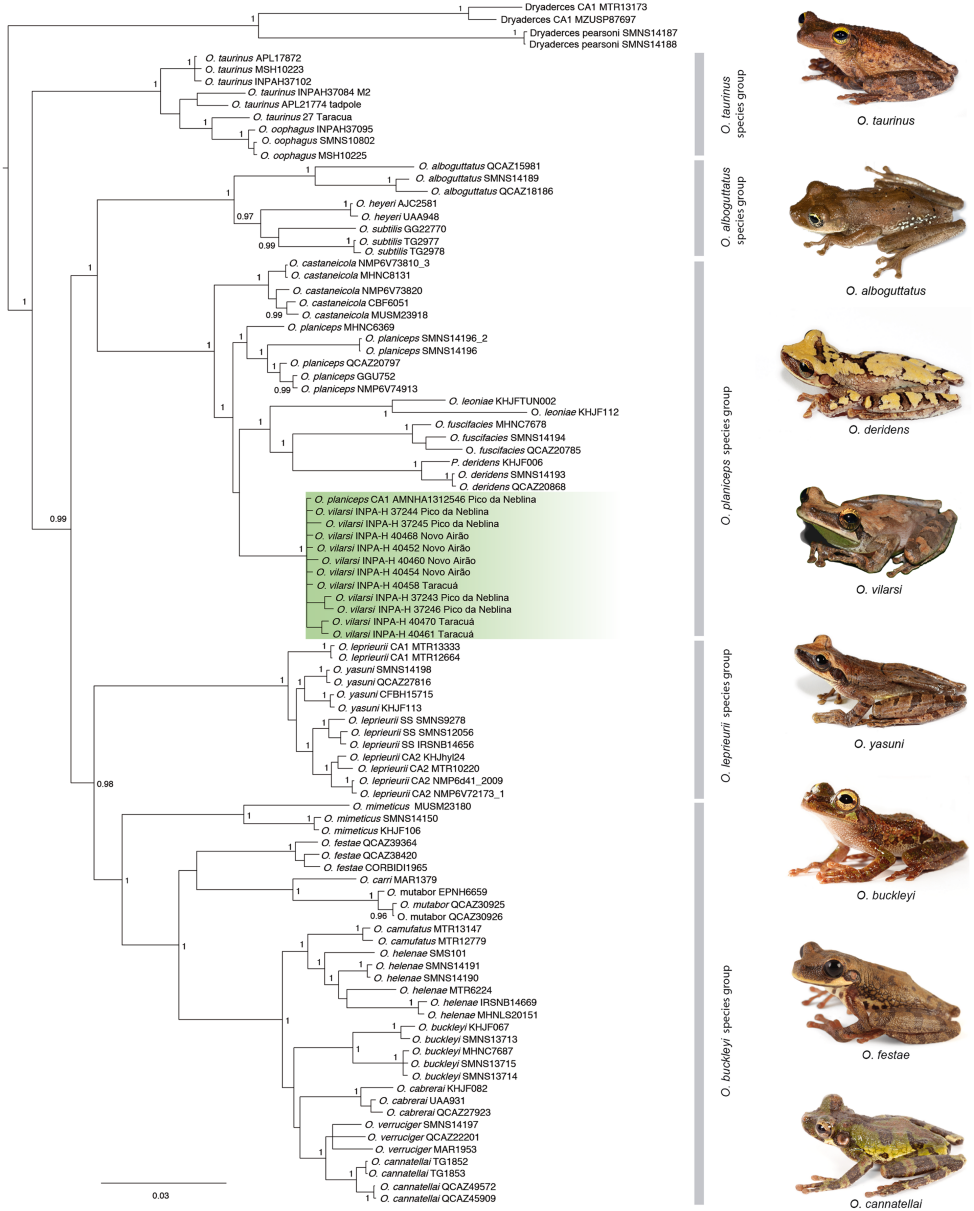
*Phylogeny* of the genus *Osteocephalus* based on five mitochondrial markers (12S, 16S, COI, CYTB, ND1; 4382 bp). Posterior probability values are depicted close to nodes (only values ≥0.95 are shown) and vertical bars denote the distinct species groups within *Osteocephalus*. Samples corresponding to *O. vilarsi* are highlighted. Photographs: Santiago Ron, www.bioweb.bio (*O. taurinus*, *O. alboguttatus*, *O. deridens*, *O. yasuni*, *O. buckleyi*, *O. festae*, *O. cannatellai*), Vinicius Tadeu de Carvalho (*O. vilarsi*).

The Bayesian phylogenetic reconstruction based on five mitochondrial markers recovered the genus *Osteocephalus* as monophyletic (Fig. 2). The five species groups previously determined within *Osteocephalus* were strongly supported, as well as their evolutionary relationships. The *O. taurinus* species group is the sister clade to a large clade containing the remaining species groups. Within this large clade, the *O. alboguttatus* species group is the sister clade to the *O. planiceps* species group. The *O. alboguttatus* + *O*. *planiceps* species group is sister to the clade containing the *O. leprieurii* and *O. buckleyi* species groups (Fig. 2). With few exceptions, the inter-species relationships were highly supported (Fig. 2).

Our phylogenetic analyses clearly confirmed the status of *O. vilarsi* as a valid species. All samples related to this species, including those from the type locality, form a strongly supported and monophyletic clade (Fig. 2). In addition, the individual from the Venezuelan slope of Pico da Neblina, previously considered a candidate species related to *O. planiceps* (Ca1_Neblina411 sensu Jungfer et al., 2013), nested within the same clade as *O. vilarsi*. Despite the extensive geographic distances between the known localities of *O. vilarsi*, the average intraspecific K2P distance within this clade was surprisingly low (ca. 0.4%; Table 2).

**Table 2:** Genetic distance (in percentages and based on the 16S rRNA mitochondrial gene) between and within species belonging to the *Osteocephalus planiceps*, *O. leprieurii* and *O. taurinus* species groups. Lower diagonal: interspecific Kimura-2-parameters (K2P). Upper diagonal: interspecific uncorrected *p*-distance. Bold diagonal numbers represent intraspecific K2P genetic distance. The taxonomy of *O. leprieurii* species group follows Jungfer et al. (2013).

Species		1	2	3	4	5	6	7	8	9	10	11	12
1	*O. vilarsi*	**0.4**	2.6	3.6	4.9	4.4	4.3	7.4	6.4	6.3	6.0	6.1	5.7
2	*O. planiceps*	2.6	**1.0**	2.6	4.0	4.1	4.6	6.9	5.6	6.7	6.7	6.8	6.5
3	*O. castaneicola*	3.7	2.7	**0.6**	4.3	4.7	5.4	7.3	6.0	7.5	7.5	7.6	7.4
4	*O. leoniae*	5.1	4.2	4.4	**3.2**	5.3	6.0	9.0	7.9	8.7	8.8	8.7	8.5
5	*O. fuscifacies*	4.6	4.3	4.9	5.6	**1.0**	5.2	8.5	7.2	7.9	7.6	8.0	7.6
6	*O. deridens*	4.4	4.8	5.7	6.3	5.4	**0.7**	8.7	7.5	9.1	9.1	9.2	9.1
7	*O. oophagus*	7.9	7.3	7.8	9.7	9.1	9.5	**0.1**	2.1	6.7	7.1	7.0	6.6
8	*O. taurinus*	6.8	5.9	6.4	8.4	7.6	8.0	2.1	**1.3**	5.3	5.6	5.6	5.1
9	*O. leprieurii* SS	6.6	7.0	7.9	9.3	8.4	9.8	7.1	5.5	**0.5**	1.9	1.2	1.5
10	*O. leprieurii* CA1	6.4	7.0	8.0	9.5	8.1	9.8	7.5	5.8	1.9	**0.0**	1.8	1.2
11	*O. leprieurii* CA2	6.4	7.3	8.1	9.4	8.5	10	7.4	5.9	1.3	1.8	**0.5**	1.4
12	*O. yasuni*	5.9	6.9	7.9	9.2	8.1	9.8	7.0	5.4	1.6	1.2	1.4	**0.6**

Contrary to previous hypotheses based on morphology, *O. vilarsi* is not close related to members of the *O. taurinus* species group. Instead, the species appears nested within the *O. planiceps* species group, where it occupies (with low support) a sister position to a strongly supported clade comprising *Osteocephalus leoniae* Jungfer & Lehr, 2001, *Osteocephalus fuscifacies* Jungfer, Ron, Seipp & Almendariz, 2000 and *Osteocephalus deridens* Jungfer, Ron, Seipp & Almendariz, 2000 (Fig. 2). Regarding the interspecific K2P genetic distances within analysed species of the *O. planiceps* species group (based on 16S gene), *O. vilarsi* presented the lowest values when compared to *O. planiceps* (2.6%), and the highest values when compared to *O. leoniae* (5.1%).

Compared to other *Osteocephalus* species sympatric with *O. vilarsi*, the average K2P genetic distances between *O. vilarsi* and members of the *O. planiceps* species group were lower than those observed between *O. vilarsi* and members of the *O. leprieurii* and *O. taurinus* groups. The average K2P genetic distance between *O. vilarsi* and members of the *O. leprieurii* group ranged from 5.9% to 6.6%, while the distance between *O. vilarsi* and members belonging to the *O. taurinus* species group were 6.8% and 7.9% (Table 2). For complete genetic distance values, see Table 2.

### *Osteocephalus vilarsi* (Melin, 1941)

*Hyla* (*Trachycephalus*) *vilarsi* Melin, 1941: p. 40, Fig. 21.

*Osteocephalus taurinus*: Bokermann (1966), Trueb & Duellman (1971).

*Osteocephalus leprieurii*: Cochran & Goin (1970); Faivovich et al. (2004); Faivovich et al. (2005); Wiens et al. (2006); Jowers, Downie & Cohen (2008); Lampo, Chacón & Nava (2009); Wiens et al. (2010); Salerno et al. (2012).

*Osteocephalus leprieurii* B: Fouquet et al. (2007).

*Osteocephalus* ‘*leprieurii’*: Moravec et al. (2009); Ron et al. (2012).

*O. vilarsi*: Jungfer (2010); Jungfer et al. (2013); Frost (2019).

*Osteocephalus planiceps*: Gordo & Neckel-Oliveira (2004); Menin et al. (2017).

*O. planiceps* (Ca1_Neblina411): Jungfer et al. (2013).

### Holotype

NHMG 488, adult female from Missão Taracuá, right bank of Uaupés River (an upper Negro River tributary), elevation ca. 100 m, Amazonas, Brazil, collected on 7 April 1924 (Melin, 1941; Caldwell, Lima & Keller, 2002). The holotype (as GNM 488) was redescribed and figured by Jungfer (2010).

### Amended diagnosis

*Osteocephalus vilarsi* is a medium-sized species (as defined by Jungfer, 2010) of the *O. planiceps* species group according to the phylogeny presented in Jungfer et al. (2013) and in the present study. The species can be diagnosed by the combination of the following characters: (1) SVL 47.5–58.4 mm in adult males and 54.6–65.3 mm in adult females; (2) frontoparietal ridges on the head; (3) truncate snout in dorsal view and rounded in lateral view; (4) skin on dorsum of adult males conspicuously tuberculated; (5) vocal slits in males; (6) vocal sac distinct, subgular, moderately expanded laterally to the area between the tympanum and forearm insertion; (7) absence of subdigital nuptial excrescences in breeding males; (8) distal subarticular tubercle on Finger IV bifid; (9) toe length I < II < III < V < IV; (10) adults exhibit white tibiofibular bones, bicoloured iris (upper part bright golden with dark brown veins and fine incomplete dark brown radiation, lower part silver to bronze with dense bold dark brown veins and/or incomplete radiation) and light subocular spot; (11) metamorphs present iris entirely bright red without black reticulation, dorsum and flanks grey with dark grey blotches and spots and absence of reddish orange blotches on hand, elbow, knees, discs, and heels; (12) tadpoles at Gosner stage 36 TL = 33.0–34.5 mm, rounded snout in dorsal view, and LKRF = 2(2)/5–6(1); (13) advertisement call composed by two (169 ± 9 ms (144–180 ms)) or three notes (276 ± 48 ms (162–337 ms)), first note always formed by two pulses, and note duration of single notes lasts 51 ± 9 ms (36–65 ms).

### Comparisons

Based on the phylogenetic relationship among *O. vilarsi* and other *Osteocephalus*, detailed interspecific comparisons were made with other members of the *O. planiceps* species group (*O. castaneicola* Moravec, Aparicio, Guerrero-Reinhard, Calderón, Jungfer & Gvoždík, 2009; *O. deridens*; *O. fuscifacies*; *O. leoniae*; *O. planiceps*). Due to historical misidentification of *O. vilarsi* with members of the *O. taurinus* (*O. taurinus*; *O. oophagus* Jungfer & Schiesari, 1995) and *O. leprieurii* (*O. leprieurii*; *O. yasuni* Ron & Pramuk, 1999) species groups, comparisons between *O. vilarsi* and these species were also provided. Characteristics of the compared species are presented in parentheses.

**Figure 3:**
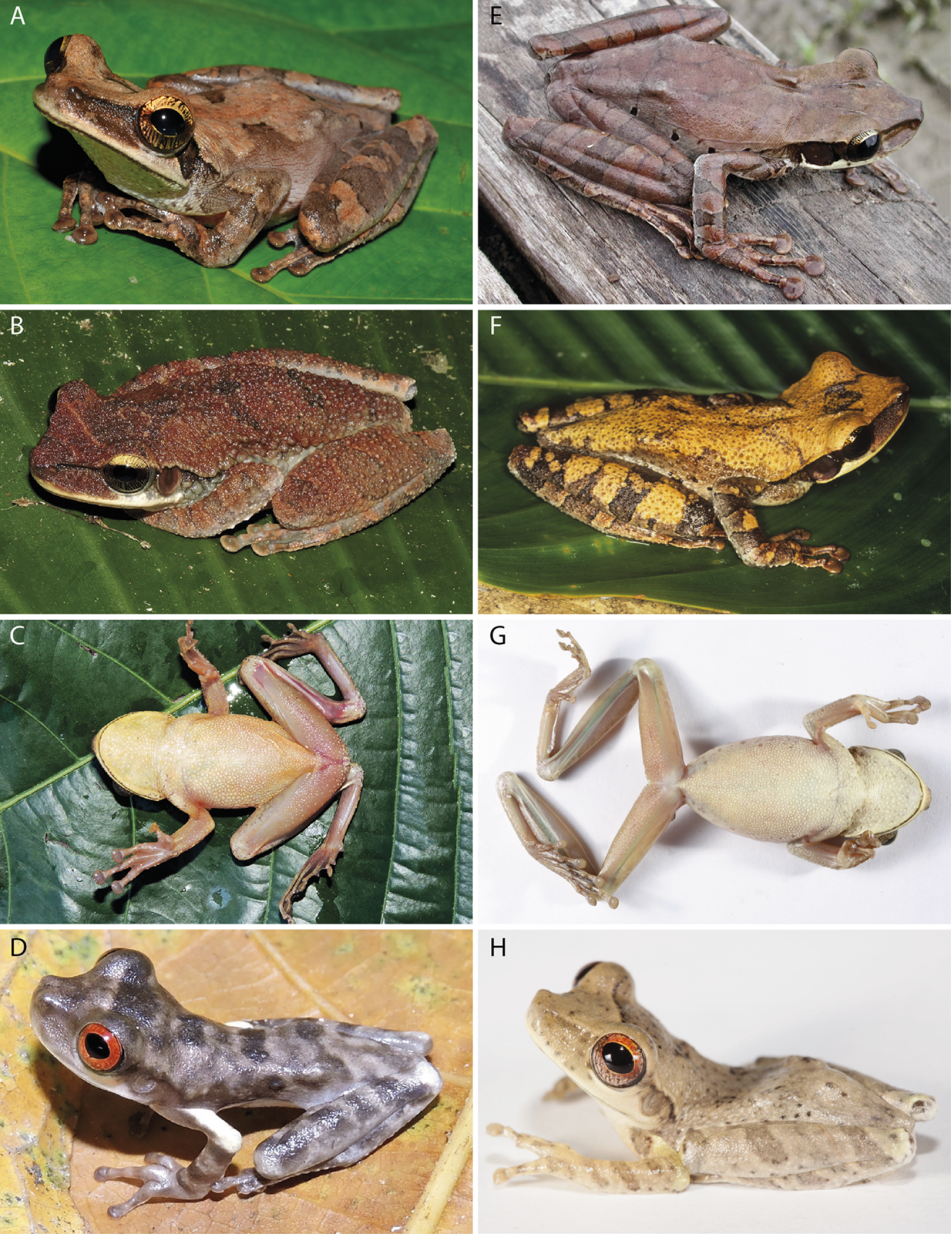
Adults and metamorphs of *Osteocephalus vilarsi* (A–D) and *Osteocephalus planiceps* (E–H). (A) Female, INPA-H 40461, from Missão Taracuá, Amazonas, Brazil. (B) Uncollected male, from RDS Rio Negro, Amazonas, Brazil. (C) Male, INPA-H 40459, from RDS Rio Negro, Amazonas, Brazil. (D) Juvenile, from RDS Rio Negro, Amazonas, Brazil. (E) Female, NMP-P6V 71264/2, from Anguilla, Loreto, Peru. (F) Male, QCAZA 148944, from Yasuní National Park, Orellana, Ecuador. (G) Female, QCAZA 44420, from Chiru Isla, North bank Napo River, Orellana, Ecuador. (H) Juvenile, QCAZA 52442, from Cuyabeno Wildlife Reserve, Sucumbíos, Ecuador. Photographs: Vinicius Tadeu de Carvalho (A), Jiří Moravec (B–C, E), Albertina Pimentel Lima (D), Santiago Ron, www.bioweb.bio (F–H).

**Figure 4:**
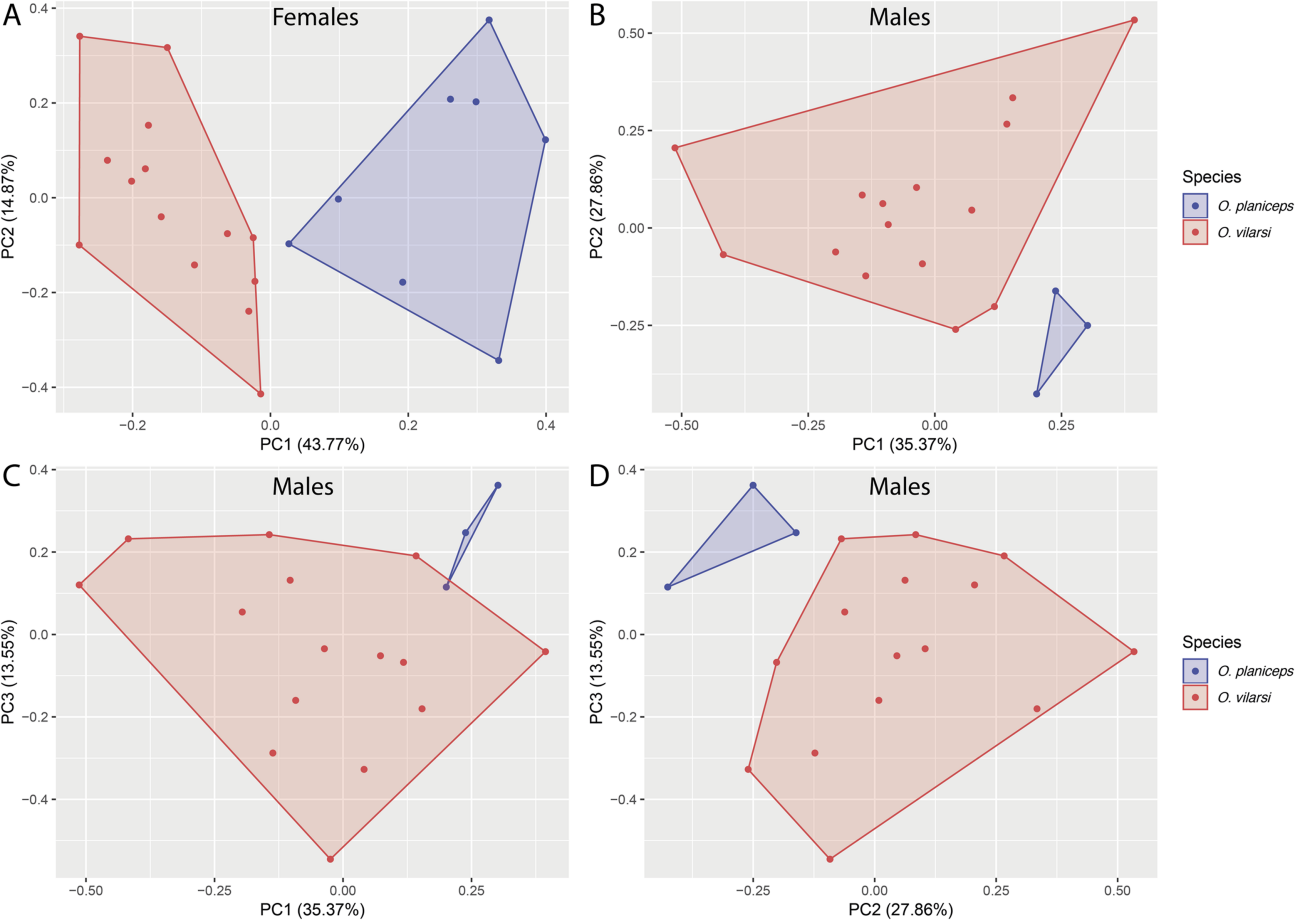
Multivariate morphometric space occupied by *Osteocephalus vilarsi* and *O. planiceps*. Principal Components Analysis (PCA) conducted with the SVL and 12 morphometric ratios of 22 females (A) and 18 males (B–D). The number of plotted PCs for each sex was determined by Broken Stick Model.

*Osteocephalus vilarsi* differs from members of the *O. planiceps* species group, as follows: from *O. castaneicola* by a truncate snout in dorsal view, the presence of vocal slits in males, vocal sac distinct and subgular, bifid distal subarticular tubercle under Finger IV (rounded snout in dorsal view, absence of vocal slits, vocal sac indistinct, single distal subarticular tubercle under Finger IV); from *O. deridens* by SVL up to 58.4 mm in adult males and 65.3 mm in adult females, conspicuously tuberculated dorsum in adult males and by white tibiofibular bones (SVL up to 34.9 mm in males and 43.7–50.6 mm in females, dorsal skin of adult males smooth, green tibiofibular bones; Jungfer et al., 2000); from *O. fuscifacies* by SVL up to 58.4 mm in adult males and 65.3 mm in adult females, a conspicuously tuberculated dorsum in adult males, the presence of a light subocular spot and by white tibiofibular bones (SVL 38.3–45.6 mm in males, adult male dorsum smooth, subocular light spot absent, green tibiofibular bones; Jungfer et al., 2000); from *O. leoniae* by the relative toe length I < II < III < V < IV, upper part of the iris bright golden with dark brown veins and fine incomplete dark brown radiation and by white tibiofibular bones (relative toe length I < II < III = V < IV, upper part of the iris yellow, green tibiofibular bones; Jungfer & Lehr, 2001); from *O. planiceps* (Fig. 3) by SVL up to 58.4 mm in adult males and 65.3 mm in adult females (SVL up to 65.4 mm in examined males and 83.2 mm in examined females: Table 3), white tibiofibular bones in life (green tibiofibular bones in life (Figs. 3C–3G) in 42 examined specimens of *O. planiceps* from Ecuador and 10 from Peru, against only one specimen with white tibiofibular bones in Ecuador). The metamorph of *O. vilarsi* (Fig. 3D) has an entirely bright red iris without black reticulation (black-reticulated iris, upper portion with red pigmentation on yellow ground and lower iris tan with red pigmentation near the pupil in metamorph of *O. planiceps*; Fig. 3H). The PCAs showed that the multivariate morphometric space occupied by females of *O. vilarsi* and *O. planieps* does not overlap and poorly overlaps in males (Fig. 4). The first two PCs retrieved by the Broken Stick Model in the PCA with females explained approximately 59% of the morphometric variation, while the first three PCs in the male analyses explained 63% of the variation (Fig. 4). The most important morphometric variables contributing to PC1 in female PCA were SVL, UEW, HW and ED. In the male PCA, the most important morphometric variables to PC1 were HW, T4W, TD and FL. See Table 4 for the contribution of all morphometric variables for other PCs. Additionally, the advertisement call of *O. vilarsi* is different from that of *O. planiceps* in structural and temporal parameters. The call duration of two-note (169 ± 9 ms (144–180 ms)) and three-note calls (276 ± 48 ms (162–337 ms)) of *O. vilarsi* is shorter (two-note calls = 229 ± 28 ms (198–259 ms), three-note calls = 402 ± 31 ms (360–465 ms) in *O. planiceps*); the first note of the advertisement call of *O. vilarsi* is always composed by two pulses (first note is mostly single (*n* = 18 or 95%) in *O. planiceps*). Single-note duration in *O. vilarsi* lasts 51 ± 9 ms (36–65 ms; *n* = 16) and is usually shorter than in *O. planiceps* (80 ± 13 ms (60–108 ms; *n* = 50)).

**Table 3:** Morphometric measurements (in mm) of *Osteocephalus vilarsi* and *O. planiceps*.

Variables	*Osteocephalus vilarsi*			*Osteocephalus planiceps*	
	Males (*n* = 15)	Females (*n* = 14)	Holotype	Males (*n* = 3)	Females (*n* = 8)
SVL	54.4 ± 2.7 (47.5–58.4)	60.1 ± 3.2 (54.6–65.3)	62.2	64.1 ± 1.2 (63.1–65.4)	72.9 ± 4.9 (66.2–83.2)
HL	18.8 ± 0.9 (17.2–20.2)	20.7 ± 1.3 (19.0–23.0)	20.1	21.3 ± 0.4 (20.9–21.6)	24.0 ± 1.8 (21.9–27.8)
HW	18.3 ± 0.9 (16.9–19.9)	20.0 ± 1.1 (18.5–21.6)	19.6	20.6 ± 0.6 (20.1–21.2)	23.4 ± 1.5 (22.1–26.8)
IOD	5.0 ± 0.5 (4.1–5.6)	5.8 ± 0.7 (4.9–6.8)	–	7.0 ± 0.2 (6.9–7.3)	8.3 ± 0.8 (7.3–9.3)
EN	5.6 ± 0.5 (4.8–6.5)	6.6 ± 0.6 (5.6–7.7)	6.2	7.0 ± 0.3 (6.8–7.3)	8.5 ± 0.8 (7.3–10.0)
ED	5.7 ± 0.3 (5.2–6.2)	6.3 ± 0.4 (5.9–7.2)	6.2	6.8 ± 0.1 (6.7–6.9)	6.8 ± 1.0 (5.7–8.5)
UEW	5.5 ± 0.4 (4.7–5.9)	5.9 ± 0.4 (5.3–6.4)	–	5.9 ± 0.2 (5.7–6.0)	6.3 ± 0.8 (5.2–7.6)
TD	4.3 ± 0.4 (3.6–4.9)	4.8 ± 0.4 (4.2–5.7)	4.8	4.9 ± 0.1 (4.8–5.0)	5.5 ± 0.3 (5.1–6.1)
3FD	3.0 ± 0.3 (2.4–3.3)	3.4 ± 0.3 (3.1–3.9)	2.3	3.7 ± 0.4 (3.4–4.1)	4.4 ± 0.6 (3.7–5.7)
THL	28.9 ± 1.7 (25.5–32.0)	32.1 ± 1.9 (28.8–34.8)	31.6	33.9 ± 1.6 (32.3–35.5)	37.4 ± 3.1 (32.4–42.3)
TL	30.6 ± 1.8 (26.5–33.5)	34.3 ± 2.2 (30.4–37.9)	34.5	36.3 ± 2.2 (34.4–38.7)	41.0 ± 2.9 (36.8–46.5)
FL	37.6 ± 2.2 (33.4–41.0)	42.4 ± 2.7 (38.2–46.2)	42.0	43.8 ± 1.2 (43.0–45.2)	49.5 ± 3.3 (44.5–55.6)
4TD	2.6 ± 0.2 (2.2–3.0)	3.0 ± 0.2 (2.7–3.4)	–	3.0 ± 0.1 (2.9–3.1)	3.9 ± 0.5 (3.1–5.0)

**Table 4:** Loadings resulting from Principal Component Analyses (PCAs) conducted with males and females of *Ostecephalus vilarsi* and *O. planiceps*. PCAs were conducted with SVL and 12 morphometric ratios (scaled). Bold numbers denote variables with the highest loadings of each Principal Component (PC). See Material and Methods for abbreviation of morphometric measurements.

Variables	Males (*n* = 18)			Females (*n* = 22)	
	PC1	PC2	PC3	PC1	PC2
SVL	0.245	**−0.325**	**0.339**	**0.355**	0.033
HL/SVL	−0.321	0.294	0.191	−0.306	−0.121
HW/SVL	**−0.392**	0.226	0.011	**−0.308**	−0.112
IOD/SVL	0.238	−0.224	**0.502**	0.307	0.333
EN/SVL	−0.249	−0.254	**0.382**	0.150	0.312
ED/SVL	0.058	**0.358**	0.239	**−0.307**	**−0.373**
UEW/SVL	−0.271	0.290	0.107	**−0.342**	−0.205
TD/SVL	**−0.343**	0.032	0.291	−0.237	0.128
F3W/SVL	−0.180	**−0.342**	0.238	0.257	−0.311
THL/SVL	−0.284	−0.251	**−0.306**	−0.269	0.176
TL/SVL	−0.129	**−0.385**	−0.265	−0.164	**0.364**
FL/SVL	**−0.335**	−0.301	−0.195	−0.231	**0.431**
T4W/SVL	**−0.358**	−0.107	0.197	0.288	**−0.347**
Variance (%)	35.37	27.86	13.55	43.77	14.87
Cumulative (%)	35.37	63.23	76.78	43.77	58.65

Furthermore, *O. vilarsi* tadpoles in Gosner stage ≥45 differ from tadpoles of other members of *O. planiceps* species groups by the presence of dark grey blotches and spots on the dorsum and flanks (dark blotches and spots absent; Moravec et al., 2009; Jungfer et al., 2013).

*Osteocephalus vilarsi* can be differentiated from all members of the *O. taurinus* species group by a bicoloured iris, with the upper part bright golden with dark brown veins and fine incomplete dark brown radiation and lower part silver to bronze with dense dark brown veins and/or incomplete radiation and by white tibiofibular bones (iris greenish gold with bold dark brown regular radiation, green tibiofibular bones in *O. taurinus* and *O. oophagus* at their type localities; Jungfer & Schiesari, 1995; Lima et al., 2006; Jungfer, 2010). Additionally, males of *O. vilarsi* differ from those of *O. taurinus* by having vocal sac distinct, subgular and moderately distensible (vocal sac subgular, paired and strongly distensible). Tadpoles of *O. vilarsi* at Gosner stage 36 differ from those of *O. oophagus* at same stage by having TL = 33.0–34.5 mm, rounded snout in dorsal view, and LKRF = 2(2)/5–6(1) (TL = 28.9 mm, nearly truncate snout in dorsal view and LKRF = 2(2)/3 in *O. oophagus* at its type locality; Jungfer & Schiesari, 1995). Moreover, recently metamorphosed *O. vilarsi* individuals differ from members of the *O. taurinus* species group by the absence of reddish orange blotches on hand, elbow, knees, discs and heels (reddish orange blotches present; Lima et al., 2006; Jungfer et al., 2013).

*Osteocephalus vilarsi* differs from all members of the *O. leprieurii* species group by the presence of frontoparietal ridges on the head and by the absence of subdigital nuptial excrescences in adult males (frontoparietal ridges absent, subdigital excrescences present in breeding males; Ron & Pramuk, 1999; Jungfer & Hödl, 2002). Moreover, males of *O. vilarsi* differ from those of *O. leprieurii* and *O. yasuni* by having vocal sac distinct, subgular and moderately distensible (vocal sac subgular, paired and distensible ventrolaterally to laterally in *O. leprieurii* and laterally in *O. yasuni*; Jungfer & Hödl, 2002; Ron & Pramuk, 1999). Additionally, recently metamorphosed *O. vilarsi* individuals diffes from those from the *O. leprieurii* species group by a grey dorsum with dark grey blotches and bright orange iris (brown dorsum with dark brown blotches and greyish iris; Jungfer & Hödl, 2002; Jungfer et al., 2013).

### Morphological description of additional adult specimens

**Figure 5:**
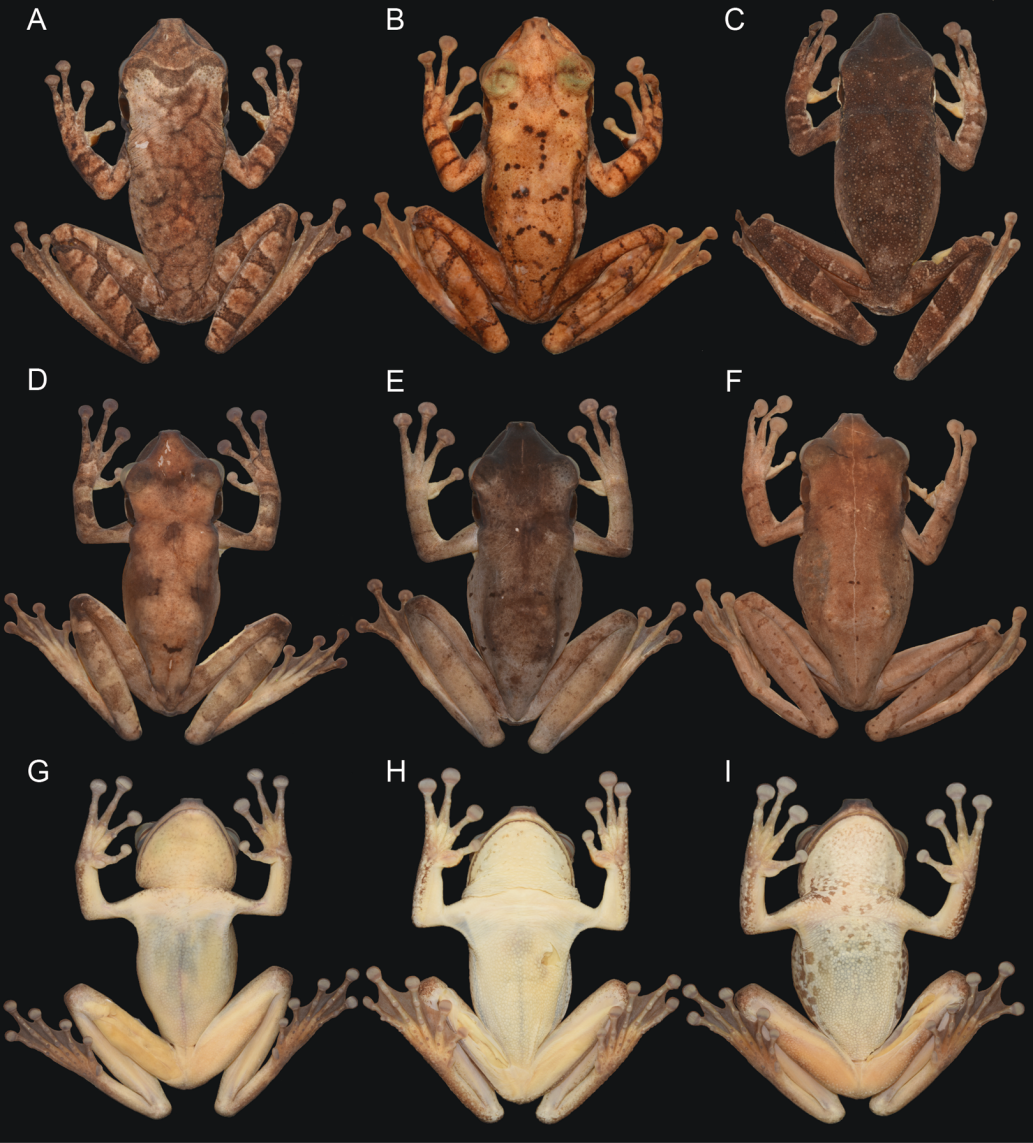
Dorsal and ventral views of adult females and males of *Osteocephalus vilarsi*. (A) INPA-H 37246, male, SVL = 55.5 mm, from the foothills of the Neblina mountain range. (B) INPA-H 40472, male, SVL = 57.2 mm, from Seringalzinho Village, Jaú National Park. (C) CZPB-AA 239, male, SVL = 58.4 mm, from the east bank of Ayuanã River, in the municipality of Santa Isabel do Rio Negro. (D) INPA-H 40461, female, SVL = 61.2 mm, from Missão Taracuá. (E) CZPB-AA 1421, female, SVL = 63.8 mm, from the northern bank of Japurá River. (F) INPA-H 40453, female, SVL = 54.6 mm, from Miratucu Lake, Jaú National Park. (G) INPA-H 40461, female. (H) INPA-H 40468, male, SVL = 55.9 mm, from RDS Rio Negro. (I) CZPB-AA 1421, female. All localities in the state of Amazonas, Brazil. Photographs and plate: Marcelo Gordo & Miquéias Ferrão.

**Figure 6:**
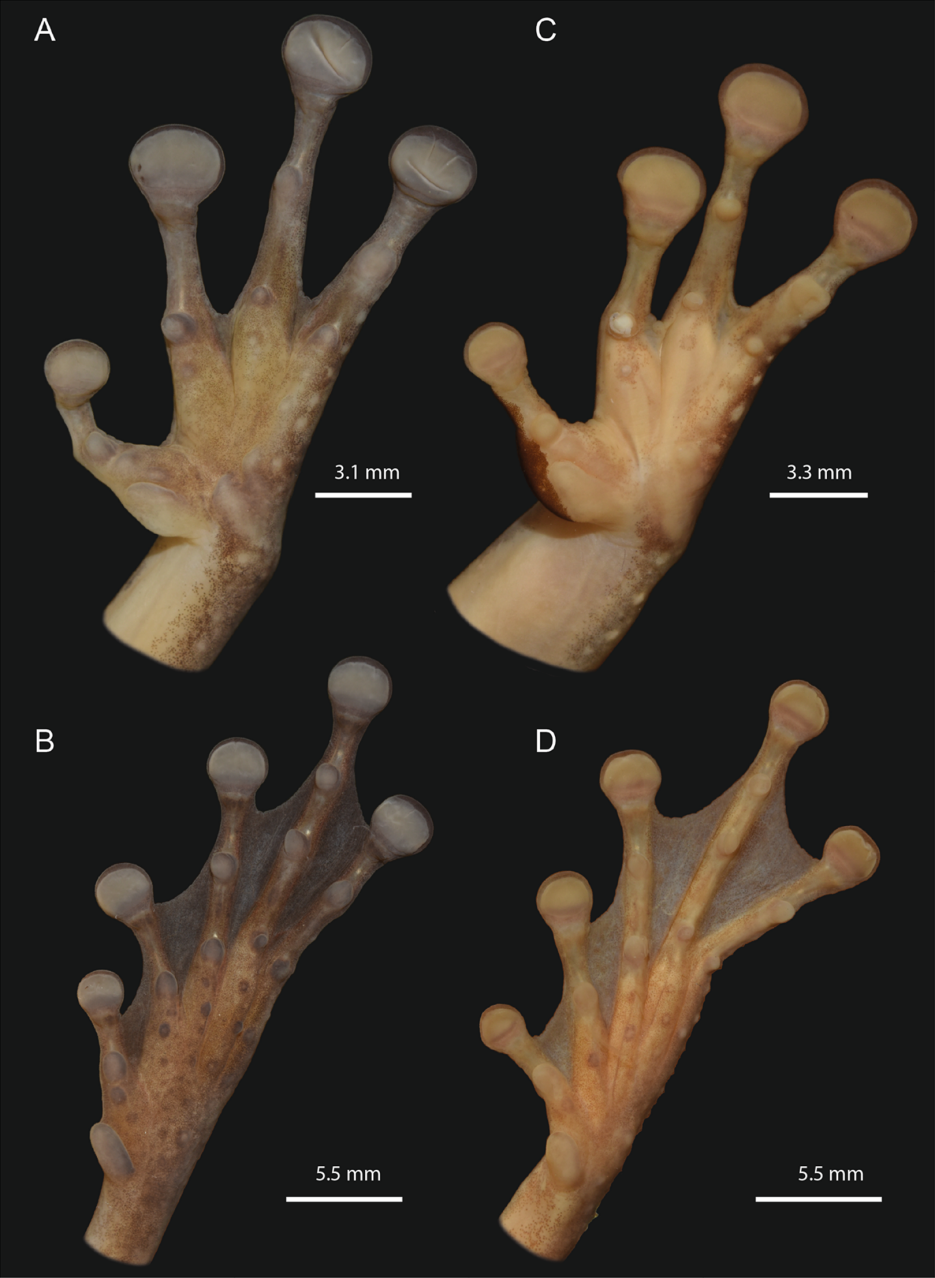
Detailed ventral views of the left hand and foot of *Osteocephalus vilarsi*. (A and B) INPA-H 40461, adult female, SVL = 61.2 mm, from Missão Taracuá (type locality). (C and D) INPA-H 40455, adult male, SVL = 54.5 mm, from Seringalzinho Village, Jaú National Park. All localities in the state of Amazonas state, Brazil. Photographs and plate: Marcelo Gordo & Miquéias Ferrão.

The morphological characteristics of 29 adult specimens of *O. vilarsi* from eight Brazilian localities are summarised as follows (adult specimen measurements are given in Table 3): medium size, SVL 47.5–58.4 mm in adult males, 54.6–65.3 mm in adult females; skin of dorsal surface with numerous minute low tubercles in females and dense protuberant tubercles bearing keratinised spinous tips in breeding males (Fig. 5); rounded snout in lateral view, truncate in dorsal view; elevated canthus rostralis, sharply angular, medially curved; deeply concave loreal region; low well-marked frontoparietal ridges in adults; large, round to oval tympanum, about 64–76% of the eye diameter, distinct tympanic annulus; markedly developed supratympanic fold; vocal slits present; vocal sac distinct, subgular, moderately expanded laterally to the area between the tympanum and forearm insertion; large, prominent, angular vomerine odontophores, narrowly separated medially, between oblique choanae, bearing 8–16 vomerine teeth each; low ulnar and tarsal tubercles present; axillary membrane present; basal webbing on hand (webbing formula II (2–2^+^)—3^+^ III (3^–^–3)—(3^–^–3) IV) (Figs. 6A and 6C); toes about three fourths webbed (webbing formula I (1^+^–1^1/2^)—( 1^1/2^–2^+^) II (1–1^+^)—(1^1/2^–2^+^) III (1–1^+^)—(2^−^–2^1/2^) IV (1^1/2^–2^−^)—(1–1^+^) V) (Figs. 4B and 4D); bifid distal subarticular tubercle under the fourth finger (except in two females from the type locality); dark keratinous nuptial excrescences covering the inner prepollex surface and extending laterally up to the thumb disc in males (Fig. 6C).

**Figure 7:**
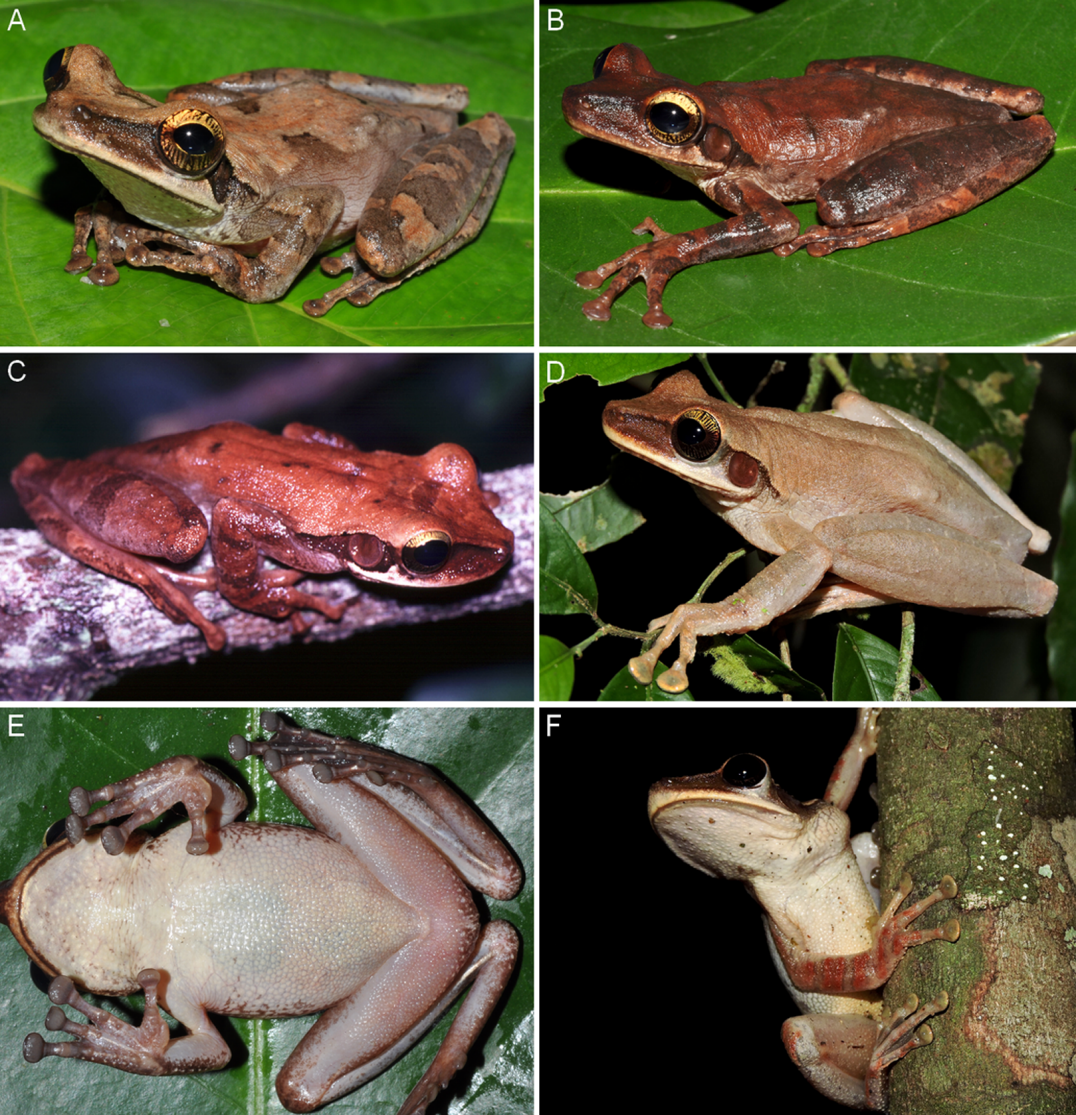
Colouration in life of females of *Osteocephalus vilarsi*. (A) INPA-H 40461, SVL = 61.2 mm, from Missão Taracuá. (B) INPA-H 40470, SVL = 65.3 mm, from Missão Taracuá. (C) Uncollected specimen from the Jaú National Park. (D) Uncollected specimen from RDS Rio Negro. (E) INPA-H 40470. (F) Uncollected specimen from RDS Rio Negro. All localities in the state of Amazonas state, Brazil. Photographs: Vinicius Tadeu de Carvalho (A, B, E), Marcelo Gordo (C), Jiří Moravec (D, F).

**Figure 8:**
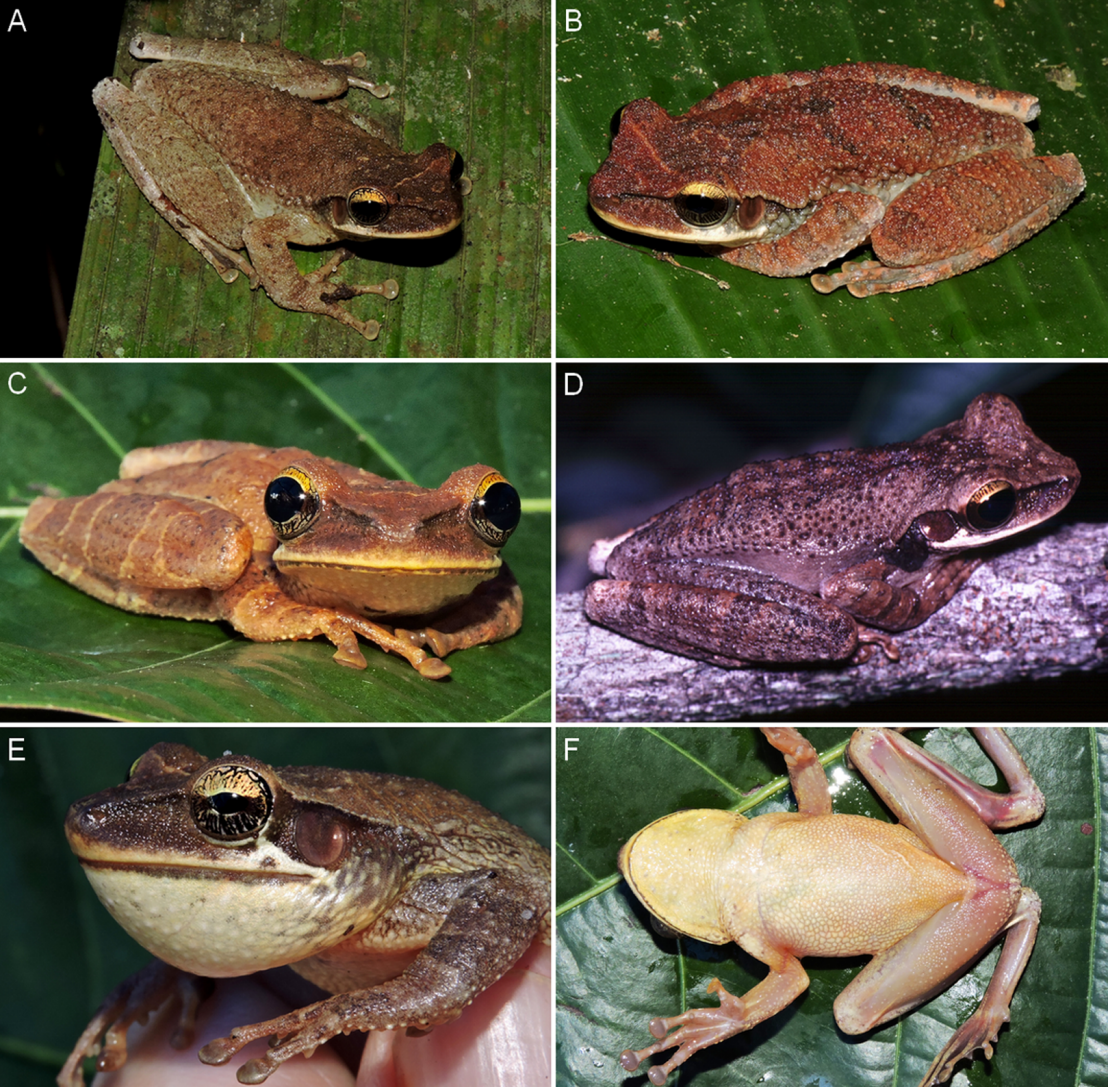
Colouration in life of males of *Osteocephalus vilarsi*. (A) INPA-H 40465, SVL = 55.5 mm, from RDS Rio Negro. (B) Uncollected, from RDS Rio Negro. (C) INPA-H 40459, SVL = 51.1 mm, from RDS Rio Negro. (D) INPA-H 40455, SVL = 54.5 mm, from the Jaú National Park, displaying the lateral projection of the vocal sac between the tympanum and arm. (E) Lateral view of a partially expanded vocal sac in male INPA-H 40465. (F) INPA-H 40459, from RDS Rio Negro. All localities in the state of Amazonas, Brazil. Photographs: Jiří Moravec (A–C, E, F), Marcelo Gordo (D).

In life (Figs. 7 and 8), adult specimens pale brown, dorsally yellowish brown to reddish brown, with or without a pattern of dark brown to black irregular markings; interorbital stripe and dark brown canthal stripe present (interorbital stripe narrower than the diameter of the eye in all specimens except for female INPA-H 40462, in which a triangular spot is formed); a narrow pale supralabial line expanding in a subocular spot; creamy to yellowish white flanks, with or without irregular brown markings or small spots; light brown hidden thigh surfaces; creamy or yellowish white throat and belly with or without diffuse small pale brown markings or spots; a narrow dark line present along the mandible; fleshy pink to slightly orange ventral thigh surfaces; bicoloured iris with a dark brown horizontal stripe, bright golden above with dark brown veins and fine incomplete dark brown radiation, silver to bronze below with dense bold dark brown veins and/or incomplete radiation (Figs. 7 and 8); white tibiofibular bones (Figs. 7E and 8F).

### Sexual dimorphism

In addition to the presence of vocal slits, vocal sac and dark keratinous nuptial excrescences in males, *O. vilarsi* also exhibits sexual dimorphism of body size and dorsal skin texture (Figs. 5–8). Adult males possess numerous protuberant spinous tubercles distributed on the dorsal surfaces of the head, body and limbs (Figs. 8A–8F). On the other hand, tubercles in adult females are minute and flat (Figs. 7A–7D).

### Advertisement call

**Figure 9:**
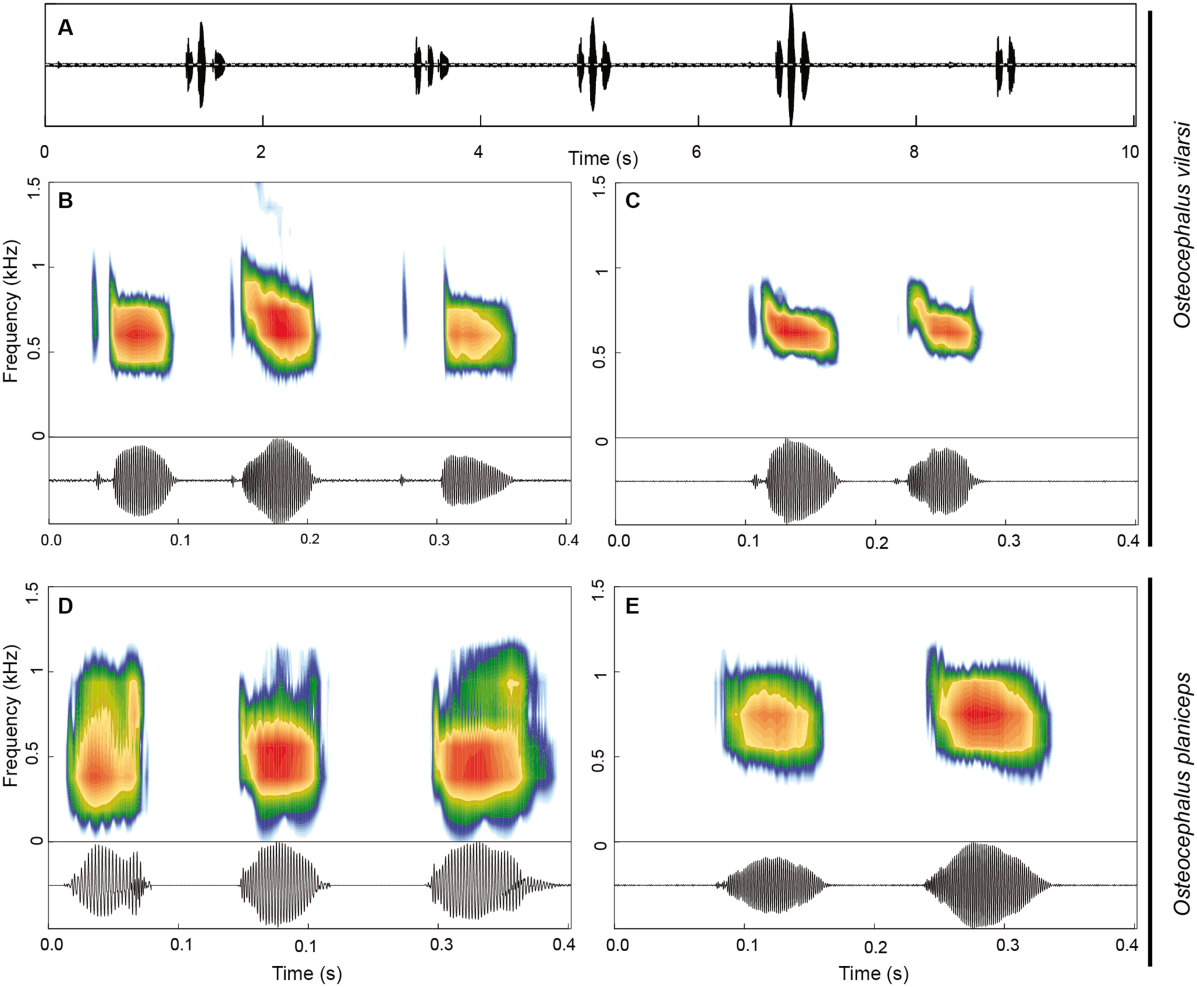
Advertisement call of *Osteocephalus vilarsi* and *O. planiceps*. (A) Oscillogram of a series of five calls of *O. vilarsi* from the Jaú National Park, Amazonas, Brazil (INPA-H 40455, SVL = 54.5 mm). Spectrograms (upper graphs) and oscillograms (lower graphs) depicting calls composed by three (B) and two (C) pulsed notes of *O. vilarsi* from the Jaú National Park (B) INPA-H 40455 and at RDS Rio Negro (C) unvouchered, Novo Airão, Amazonas, Brazil. Spectrograms (upper graphs) and oscillograms (lower graphs) depicting calls of *O. planiceps* composed by three (D) and two (E) single notes recorded at Pompeya Sur (D), Orellana, Ecuador and at Reserva Biológica Jatun Sacha (E), Napo, Ecuador.

The advertisement call of *O. vilarsi* (Figs. 9A–9C) exhibits a call duration of 211 ± 61 ms (144–337; *n* = 23) and consists of notes emitted in pairs (*n* = 14; common in less motivated males) or trios (*n* = 9; common in motivated males). The first note of calls is always formed by a pair of pulses (*n* = 23). The second and third notes consist of one (*n* = 16) or two pulses (*n* = 16). Note duration of pulsed and unpulsed notes ranges from 36 to 116 ms (59 ± 13; *n* = 55), while the note duration of pulsed notes is 45–116 ms (63 ± 13; *n* = 39) and of the single note is 36–65 ms (51 ± 9; *n* = 16). The inter-note interval lasts 39–73 ms (54 ± 10; *n* = 32). The pulse duration of the first pulse in pulsed notes is shorter 3–10 ms (6 ± 1; *n* = 39) than that of the second pulse 18–75 ms (47 ± 12; *n* = 39). Pulses are interrupted by a short inter-pulse interval lasting 3–37 ms (9 ± 7; *n* = 39). The first pulse is tonal while the second pulse contains 1–4 (2 ± 1) harmonics. The dominant frequency is located in the fundamental harmonic. The overall dominant frequency is 474–948 Hz (624 ± 71; *n* = 91). The first pulse is characterised by a dominant frequency of 560–948 Hz (685 ± 59; *n* = 36), whereas the dominant frequency of the second pulse is slightly lower, at 474–689 Hz (584 ± 44; *n* = 55). Bioacoustic parameters of the advertisement call of *O. vilarsi* and *O. planiceps* (Figs. 9D–9E) are presented in Table 5.

**Table 5:** Acoustic parameters of the advertisement call of *Osteocephalus vilarsi* and *O. planiceps*.

Traits	*O. vilarsi* (*n* = 3)	*O. planiceps* (*n* = 3)
Overall call duration (ms)	211 ± 61 (144–337; *n* = 23)	369 ± 134 (108–608; *n* = 19)
Number of notes	2.4 ± 0.5 (2–3; *n* = 23)	2.8 ± 0.8 (1–4; *n* = 19)
Two-note call duration (ms)	169 ± 9 (144–180; *n* = 14)	229 ± 28 (198–259; *n* = 5)
Three-note call duration (ms)	276 ± 48 (162–337; *n* = 9)	402 ± 31 (360–465; *n* = 10)
Four-note call duration (ms)	–	580 ± 33 (544–608; *n* = 3)
Overall note duration (ms)	59 ± 13 (36–116; *n* = 55)	79 ± 14 (58–108; *n* = 53)
Single note duration (ms)	51 ± 9 (36–65; *n* = 16)	80 ± 13 (60–108; *n* = 50)
Pulsed note duration (ms)	63 ± 13 (45–116; *n* = 39)	93 ± 6 (87–97; *n* = 39)
Inter-note interval (ms)	54 ± 10 (39–73; *n* = 32)	80 ± 20 (34–155; *n* = 34)
First-pulse duration (ms)	6 ± 1 (3–10; *n* = 39)	15 ± 4 (12–20; *n* = 3)
Second-pulse duration (ms)	47 ± 12 (18–75; *n* = 39)	61 ± 4 (58–65; *n* = 3)
inter-pulse Interval (ms)	9 ± 7 (3–37; *n* = 39)	17 ± 2 (15–19; *n* = 3)
Dominant Frequency (Hz)	624 ± 71 (474–948; *n* = 9)	639 ± 198 (323–1162; *n* = 53)

**Note:**

Values are presented as mean ± standard deviation (minimum–maximum). Abbreviation: Hz, hertz; ms, millisecond; *n*, sample size.

### Tadpoles

**Figure 10:**
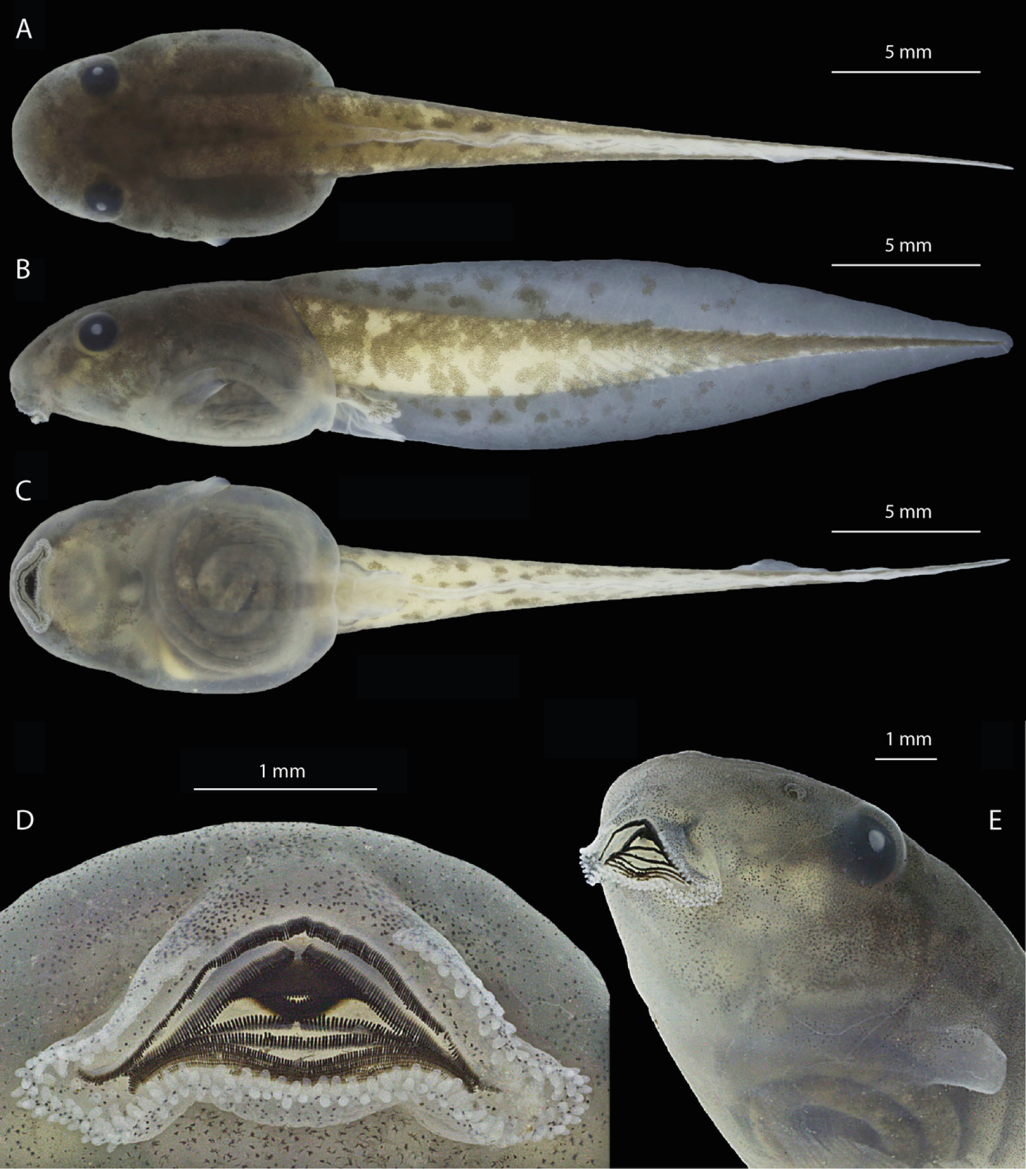
Preserved tadpole of *Osteocephalus vilarsi* at developmental Gosner stage 36 (INPA-H 40471). Specimen collected at RDS Rio Negro, municipality of Novo Airão, Amazonas state, Brazil. Dorsal (A), lateral (B) and ventral (C) views. Detailed views of the oral disc (D) and body (E). Photographs: Jeni Lima Magnusson.

The following description is based on five tadpoles at Gosner stage 36 (INPA-H 40471) collected at RDS Rio Negro, Novo Airão, Amazonas, Brazil. Total length 34.0 ± 0.6 mm (33.0–34.5 mm). Body compressed in lateral view, ovoid in dorsal view; maximum body height lower than the maximum tail height; body length 44–50% of the tail length. Rounded snout in both dorsal and lateral views (Figs. 10A and 10B). Nostrils located dorsolaterally and directed anterolaterally; circular opening. Internarial distance narrower than the interorbital distance (IND/IOD = 0.69–0.74). Eyes located and directed dorsolaterally. Oral disc (Figs. 10D and 10E) located and directed anteroventrally, large (ODW/BW = 0.43–0.47), with protuberant labia; presence of marginal papillae gap in the medial region of the upper labium present (32–35% of oral disc width); cylindrical and long papillae; one to three rows of papillae on the central and posterior portions of the upper labium; two rows of papillae on the entire lower labium, except for the corner (three rows). Slender upper jaw sheath, finely serrated and arc-shaped; lower jaw sheath as slender and finely serrated as the upper one, V-shaped. Labial keratodont row formula (LKRF) 2(2)/5–6(1); A-2 gap small but larger than the P-1 gap. Single and sinistral spiracle; inner wall free from the body; in lateral view, the spiracle surpasses the outline of the lower margin of the tail muscle in the distal portion. Dextral vent tube, attached to the ventral fin, right wall displaced dorsally. Dorsal and ventral fin similar in height; the ventral fin does not surpass the lower margin of body; flagellum absent.

Measurements (in mm) are as follows, expressed as means ± standard deviation (range): TL, 34.0 ± 0.6 (33.0–34.5); BL, 10.8 ± 0.2 (10.5–11.0); BW, 7.3 ± 0.2 (7.0–7.6); TAL, 23.2 ± 0.7 (22.0–24.0); TMH, 2.4 ± 0.1 (2.3–2.5); MTH, 6.5 ± 0.1 (6.3–6.7); IOD, 4.4 ± 0.2 (4.0–4.5); IND, 3.2 ± 0.1 (2.8–3.4); TMW, 3.2 ± 0.1 (3.0–3.3); ODW, 3.3 ± 0.1 (3.1–3.4); ODG, 1.0 ± 0.0 (1.0–1.0).

### Tadpole and metamorph colouration

**Figure 11:**
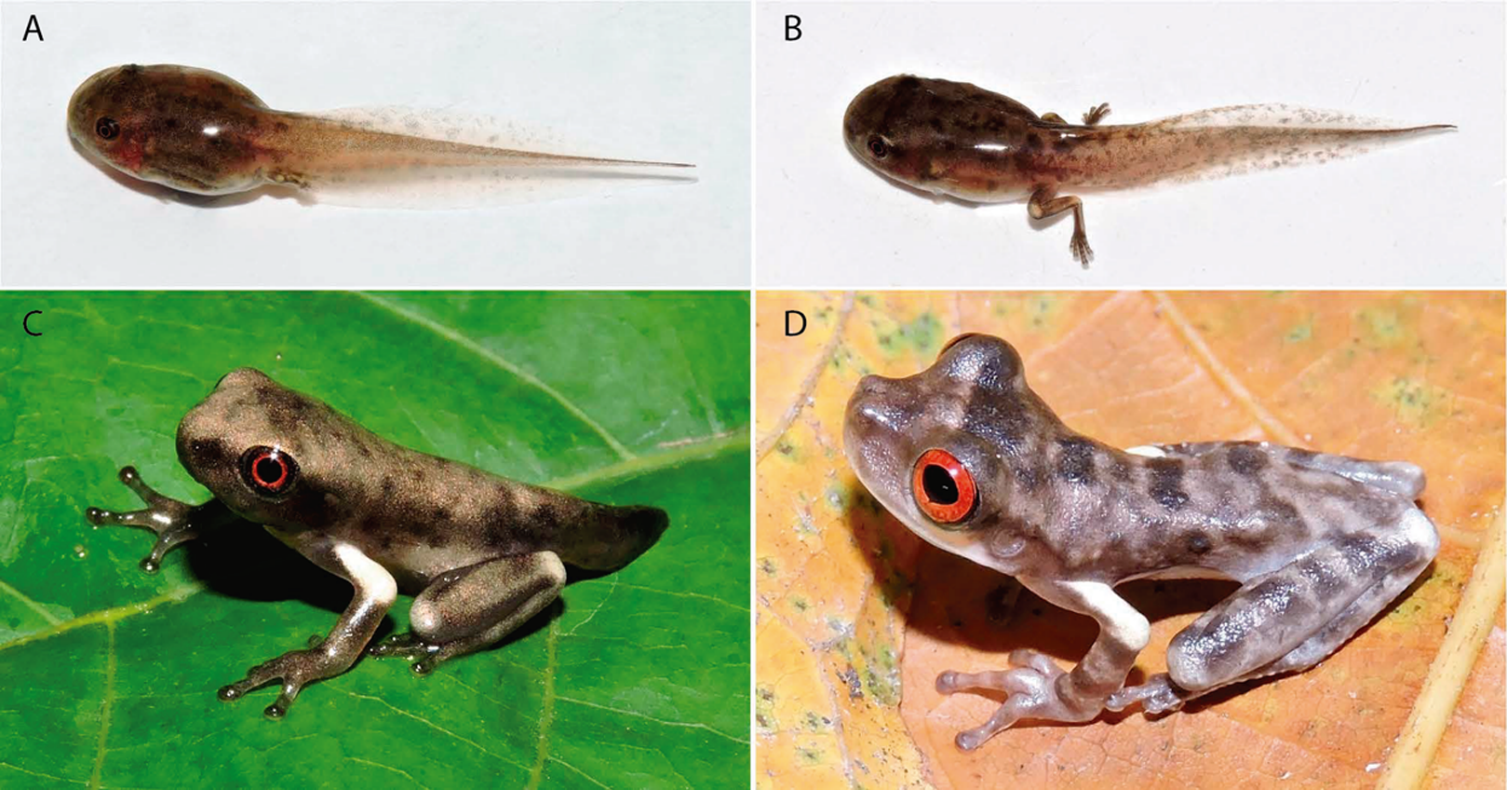
Ontogenetic colouration changes of tadpoles and metamorphs of *Osteocephalus vilarsi*. Specimens collected at RDS Rio Negro, municipality of Novo Airão, Amazonas, Brazil. (A) Tadpole at Gosner stage 36. (B) Tadpole between Gosner stage 39 and 40. (C) Metamorph at Gosner stage ~45. (D) Recently metamorphosed juvenile. Photographs: Jiří Moravec (A–C), Albertina Pimentel Lima (D).

In life, tadpoles at Gosner stage 36 display a light brown dorsum with irregularly distributed dark brown blotches (Fig. 11A); a dark brown band from the nostril to the anterior portion of the orbits; a black iris with a red ring around pupil. Translucent venter. Dorsal portion of the tail muscle bronze; ventral portion pinkish cream; fins translucent with light brow blotches. Tadpoles at Gosner stage 39–40 are similar in colour pattern (Fig. 11B), except for the dorsum colour and fin blotches (that become darker) and by the presence of a large cream blotch on the knee and heel. Tadpoles at Gosner stage 45 display a silvery cream dorsum with dark and light grey blotches and spots (Fig. 11C); a dark grey band covering the lateral of head, from the snout to the posterior portion of the tympanum and on the upper lips; black iris with a red ring around pupil; grey fingers, hands, forearms, toes and feet; white upper arm; white blotch on the knee and heel; light grey groyne; dark grey tail; white venter.

In life, metamorphs present a grey dorsum with dark grey blotches (Fig. 11D); a large dark grey band on the interorbital region. White subocular region, extending to the anterior infratympanic area; a dark stripe covering supratympanic region, from the posterior corner of the eyes to the arm insertion to the body. Bright red iris and black oval pupil. Limbs grey, except for the upper arm (white); four transversal dark grey stripes on the thigh; three horizontal dark grey stripes on the forearm and tarsus; light grey blotch on knee; white blotch on the heel. Venter white.

### Reproductive behaviour

**Figure 12:**
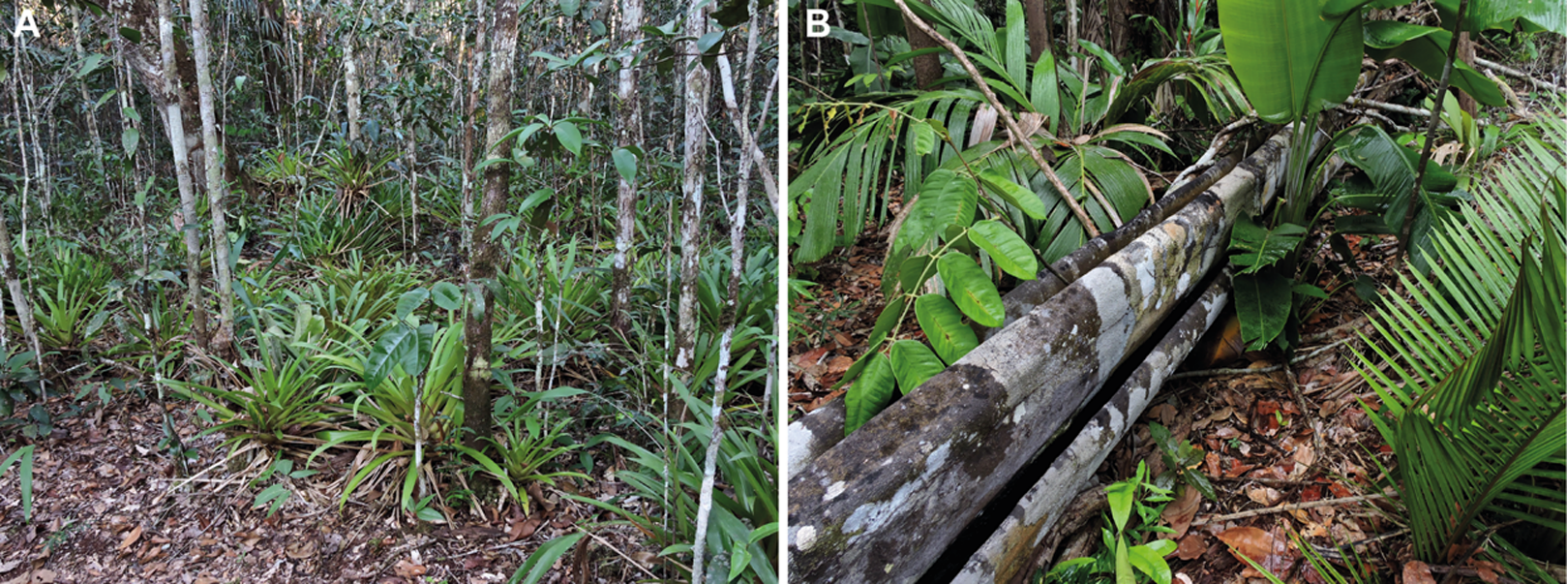
Habitat of *Osteocephalus vilarsi* at RDS Rio Negro, municipality of Novo Airão, Amazonas, Brazil. (A) A typical *O. vilarsi* habitat in an ecotone between a white-sand forest and dense rainforest in September 2018. (B) An *O. vilarsi* breeding place in a disturbed forest in October 2017. Males were calling on plants close to trunk ridges filled with rainwater. Photographs: Jiří Moravec.

Two *O. vilarsi* calling males were observed at RDS Rio Negro (municipality of Novo Airão, Negro–Solimões interfluve) during 24–26 October 2017. The first male (not collected) called from the same place for three consecutive nights. It occupied a calling place on a horizontal leaf of herbaceous plant ca. 60 cm above the ground. The plant was growing close to a small phytotelma formed by trunk ridges of a large fallen tree (Fig. 12B). The phytotelma was approximately at the same height above ground as the calling place. The second male was found calling on vegetation ca. 40 cm above the ground at a small temporary forest puddle. Both breeding places were also occupied by calling males of *Rhinella* sp. (*Rhinella margaritifera* species group). Tadpoles from RDS Rio Negro were found in a shallow puddle not connected to a stream in a secondary white-sand forest. Specimens from the Jaú National Park were collected and observed in reproduction (May 2000) on the ground or up to 1 m high in shrub branches in open white-sand forests known as *campina* (canopy below 10 m), near or above temporary puddles. Some calling males presented territorial behaviour by altering their vocalisation in response to a playback (not recorded). Based on those notes, it appears that *O. vilarsi* can breed in small water body sources in closed forests (e.g. small temporary puddles, phytotelmata), as well as in open forests.

### Distribution and habitat

Until now, *O. vilarsi* was recorded in localities inside the interfluve between the Negro and Solimões rivers, as well as at the left bank of the upper Negro River (Figs. 1A and 1B). Such a wide geographic range covering approximately 212,000 km^2^ indicates that *O. vilarsi* is widely distributed across the extreme North-western Brazil (state of Amazonas) and the adjacent Southern Venezuela. The western most known locality is ca. 40 km from the Colombian border. Therefore, its occurrence can be expected in Colombian territory.

During field surveys in the vicinity of the municipality of Novo Airão, *O. vilarsi* was recorded both in relatively undisturbed forests and in heavily altered habitats adjacent to communities and farmlands. It was found both in the semi-open white-sand forest known as *campinarana* (canopy below 20 m; Fig. 12A) and in high closed forests (canopy above ca. 20 m; Fig. 12B). No individuals were found in the open white-sand vegetation known as *campina* (canopy below 10 m). After dusk, individuals were found perched on vegetation (mostly on narrow vertical trunks of smaller trees) ca. 50–200 cm above the ground. In two cases, adult individuals were found hidden in open vertical plastic tubes, used for the delimitation of INPA study plots. Other hylid species found in sympatry with *O. vilarsi* at Novo Airão include *Boana boans* (Linnaeus, 1758), *B*. aff. *geographica*, *B. lanciformis* (Cope, 1871), *Dendropsophus minutus* (Peters, 1872), *O. taurinu*s (in low abundance compared to *O. vilarsi*), *Scinax ruber* (Laurenti, 1768), *Scinax* sp., and *Trachycephalus cunauaru* Gordo, Toledo, Suárez, Kawashita-Ribeiro, Ávila, Morais & Nunes, 2013.

Several non-reproductive specimens of *O. vilarsi* at Jaú National Park were observed in both flooded and unflooded rainforests (known as *igapó* and *terra firme* forests, respectively; canopy above ca. 20 m), *campinarana* (canopy below 20 m), and *campina* (comprising shrubs and exposed sand). Specimens were perched on horizontal branches of small trees or on vertical trunks. Other hylid species found in sympatry with *O. vilarsi* at the Jaú National Park are listed in Neckel-Oliveira & Gordo (2004).

Similarly, individuals recorded during the field survey at the Brazilian foothills of the Pico da Neblina, close to the Venezuelan border, were found perched on horizontal branches of small trees, in an ecotonal white-sand forest (*campinarana*) with a low canopy and several small temporary puddles. In this region, *O. vilarsi* occurs in sympatry with the hylids *Boana* aff. *cinerascens*, *B. lanciformis*, *Dendropsophus minutus*, *D. tintinnabulum* (Melin, 1941), *Osteocephalus* aff. *taurinus*, *Scinax ruber* and *S. cruentommus* (Duellman, 1972).

In fact, all individuals of *O. vilarsi* analysed in this study were recorded in localities covered by white-sand forest, or in close vicinity. Such an association indicates an ecological preference of this species to this particular and threatened type of Amazonian forest. In fact, the estimated distribution of *O. vilarsi* presented herein mostly coincides with the occurance of this forest type inside the Amazon in North-western Brazil and adjacent Colombia and Venezuela (Fig. 1A).

## Discussion

The results of the phylogenetic analysis based on five mitochondrial markers are in good agreement with the previous broader phylogenetic hypotheses proposed for *Osteocephalus* by Ron et al. (2012) and Jungfer et al. (2013). Monophyly of the five species groups was strongly supported. The main difference between the present study and previous phylogenetic analyses lies in the topology of the species groups. Whereas Ron et al. (2012) and Jungfer et al. (2013) recovered the *O. planiceps* species group as sister to the *O. leprieurii* species group, our analyses placed this group in a sister position to the *O. alboguttatus* species group. Different topologies concerning these three phylogenetic hypotheses may reflect differences in specimen sampling, analysed genes and amount of missing data from each dataset.

Our phylogenetic and morphological analyses revealed *O. vilarsi* to be a valid species belonging to the *O. planiceps* species group. It is interesting however, that one individual of *O. vilarsi* (AMNH 1312546, Venezuelan slopes of the Pico da Neblina mountain range) was already recognised at the specific level in most of the previous phylogenetic hypotheses concerning the genus. Due to the absence of other voucher specimens and lack of comparative morphological data, the individual from the Venezuelan slope of the Pico da Neblina was wrongly associated with other *Osteocephalus* (e.g. *O. leprieurii*, *O*. ‘*leprieurii’*, or *O. planiceps* CA1; Wiens et al., 2006; Moravec et al., 2009; Wiens et al., 2010; Ron et al., 2012; Salerno et al., 2012; Jungfer et al., 2013).

The apparent strong ecological association of *O. vilarsi* to white-sand ecosystems (e.g. primary and secondary white-sand forests, as well as ecotonal zones between white-sand forests, *igapó* and *terra firme* forests) represents a rarely reported example of such an association in Amazonian anurans. To the best of our knowledge, a similar ecological association is known only in the case of the casque-headed frog *Aparasphenodon venezolanus* (Mertens, 1950), whose distribution partially overlaps with that of *O. vilarsi* at Jaú National Park (De Carvalho et al., 2018). Despite rarely reported in anurans, such a specific association is widely known and studied in Amazonian birds, which present highly endemic white-sand assemblages (Adeney et al., 2016; Borges et al., 2016).

As the white-sand forests cover large but patchily distributed portions of the upper and middle Negro River basin (Adeney et al., 2016), we expect *O. vilarsi* to present a similar distribution pattern across this area. White sand ecosystems belong to the most sensitive vegetation types in Amazonia (Adeney et al., 2016), and the effects of their exploration and alteration on the dynamics of *O. vilarsi* populations in different parts of the Amazon are still unknown.

*Osteocephalus vilarsi* is a remarkable case of a widely distributed Amazonian anuran species, which has been overlooked for decades. Its rediscovery illustrates how an integrative approach to the study of Amazonian amphibians is vital. Our extensive examination of morphological, molecular, geographical, ecological, and bioacoustical data, combined with the revision of museum collections has updated and extended the geographic range of the species (previously known only from its type locality) to an area covering over 200,000 km^2^ at the Negro-Solimões Interfluve and surrounding Brazilian and adjacent Venezuelan areas (the species is also expected to occur in adjacent Colombia). The scientific knowledge concerning *O. vilarsi* should rapidly increase, since the data reported herein (including morphological variation in adults, juveniles and tadpoles and advertisement call characteristics) should serve as a basis for further identification of individuals belonging to this species.

## Conclusion

*Osteocephalus vilarsi* (Melin, 1941) has been rediscovered 75 years after its description. Based on morphological and molecular analyses, *O. vilarsi* is replaced from the *O. taurinus* species group to the *O. planiceps* species group. Morphological variation of adult, subadult and juvenile specimens, morphology of tadpoles, advertisement call, and natural history of *O. vilarsi* are described for the first time. Biogeographic data demonstrate that *O. vilarsi*, previously known only from its type locality, is widely distributed across the interfluve between the Negro and Solimoês rivers in North-western Brazil and adjacent southern Venezuela. The species displays strong ecological association to white-sand ecosystems (primary and secondary white-sand forests).

## Appendix I

Additional specimens examined. Symbols: * = photograph.

*Osteocephalus buckleyi*: BOLIVIA: Pando: Santa Crucito (NMP-P6V 73945), Pando (CBF 1262, CBF 2150–51).

*Osteocephalus cabrerai*: PERU: Loreto: 21 km W of Iquitos (NMP-P6V 71144/1–2).

*Osteocephalus castaneicola*: BOLIVIA: Pando: San Antonio (CBF 6051 (holotype), NMP-P6V 73810/1–3 (paratopotypes), NMP-P6d 28/2009).

*Osteocephalus deridens*: PERU: Loreto: Anguilla (NMP-P6V 71263), 35 km SW of Iquitos (NMP-P6V 71262/1–5).

*Osteocephalus fuscifacies*: ECUADOR: Napo: Aliñahuí, 5 km W of Ahuano (ZFMK 68660* (paratype)).

*Osteocephalus mimeticus*: PERU: Huánuco/Ucayali: El Boquerón del Padre Abad (ZFMK 33352, ZFMK 36319).

*Osteocephalus mutabor*: ECUADOR: Napo: Río Chaloyacu on Carretera Narupa – Coca (ZFMK 66237* (paratype)).

*Osteocephalus oophagus*: BRAZIL: Amazonas: Reserva Forestal Adolfo Ducke (ZFMK 57137*–*38* (paratypes)).

*Osteocephalus planiceps*: ECUADOR: Napo: Puerto Misahaulli at La Cruz Blanca (MCZ-A 111190, MCZ-A 111188), Limoncocha (MCZ-A 98000, MCZ-A 98019, QCAZA 63543*), Laguna Taracoa (MCZ-A 97755); Orellana: Yasuni National Park (QCAZA 14844*, QCAZA 20797*, QCAZA40987*, QCAZA 51085*, QCAZA 55257*, QCAZA 55297–8*, QCAZA 55364*, QCAZA 55378*, QCAZA 55380*, QCAZA 64119–21*, QCAZA 64125–31*), Napo River (QCAZA 43891*, QCAZA 44420*); Pastaza: Lorocachi (QCAZA 55857*, QCAZA 55874*, QCAZA 55879*, QCAZA 55881*, QCAZA 55908*, QCAZA 55924*, QCAZA 55999*, QCAZA 56011*, QCAZA 56017*, QCAZA 56046*, QCAZA 56061*, QCAZA 56624*), Campo Villano (QCAZA 38702*), Pupalyacu (QCAZA 56624*); Morona Santiago: Comunidad Jempekat (QCAZA 54404*), San José de Morona (QCAZA 73720*), Marantian Wildlife Reserve (QCAZA 75756*); Sucumbíos: Cuyabeno Wildlife Reserve (QCAZA 37786*, QCAZA 52426*), Sacha Lodge (QCAZA 42288*), La Selva Amazon Ecolodge (QCAZA 44060*). PERU: Loreto: Anguilla (NMP-P6V 71264/1–2), Tarapoto surr., 21 km W of Iquitos (NMP-P6V 71204/1–2), Puerto Almendras (NMP-P6V 71174/1–5), Nauta (ANSP 11399, holotype*). Ucayali: Regional Conservation Area Imiría (NMP-P6V 74913).

*Osteocephalus subtilis*: BRAZIL: Acre: Cruzeiro do Sul (MZUSP 60561* (holotype)).

*O. taurinus*: BOLIVIA: Pando: Nacebe (NMP-6V 72172/1*–*2), Pando (CBF 1281, 1300*-*02, 2147*-*48, 43333). PERU: Loreto: Puerto Almendras (NMP-P6V 71184); Ucayali: Regional Conservation Area Imiría (NMP-P6V 74441).

*O. vilarsi*: BRAZIL: Amazonas: Missão Taracuá (INPA-H 40458, INPA-H 40470, INPA-H 40461, GNM 488 (holotype; photographs and data available in Jungfer, 2010)); Rio Negro Sustainable Development Reserve (INPA-H 40468, INPA-H 40456, INPA-H 40459, INPA-H 40465, INPA-H 40460, INPA-H 40462, INPA-H 40454, INPA-H 40452, INPA-H 40471 (tadploes)); Seringalzinho Village, Jaú National Park (INPA-H 40464, INPA-H 40472, INPA-H 40467, INPA-H 40455, INPA-H 40463, INPA-H 40466, INPA-H 40473); 5 km south Seringalzinho Village, Jaú National Park (INPA-H 10940, INPA-H 10942, INPA-H 10939, INPA-H 10944); Miratucu Lake, Jaú National Park (INPA-H 40469, INPA-H 40453, INPA-H 40457); east bank of Ayuanã River (CZPB-AA 239); northern bank of Japurá River (CZPB-AA 1421); Pico da Neblina National Park (INPA-H 37243–4, INPA-H 37246, INPA-H 37245).

*Osteocephalus yasuni*: ECUADOR: Napo: Yasuní Scientific Research Station (QCAZA 11336* (holotype), QCAZA 10879* (paratopotype), QCAZA 11329* (paratopotype)). PERU: Ucayali: Regional Conservation Area Imiría (NMP-P6V 74442/1–4).

## Supplemental Information

10.7717/peerj.8160/supp-1 Supplemental Information 1 Sampling localities of *Osteocephalus vilarsi* in the Amazonas state, Brazil. Symbols: * = specimens previously published as *Osteocephalus planiceps*. References are available in the main text.

10.7717/peerj.8160/supp-2 Supplemental Information 2 Samples of the *Osteocephalus* and *Dryaderces* genera used for the phylogenetic analyses. See text for abbreviations.

10.7717/peerj.8160/supp-3 Supplemental Information 3 Morphometric measurements of specimens of *Osteocephalus vilarsi* and *O. planiceps* used in the Principal Component Analyses (PCA). Abbreviations are described in the main text.

## Additional Information and Declarations

### DNA Deposition

The following information was supplied regarding the deposition of DNA sequences: The sequences are accessible at GenBank: MK572692 to MK572704.

### Data Availability

The following information was supplied regarding data availability: The raw measurements are available in Table S3.

## References

[ref-1] Adeney JM, Christensen NL, Vicentini A, Cohn-Haft M (2016). White-sand ecosystems in Amazonia. Biotropica.

[ref-2] Altig R, McDiarmid RW, McDiarmid RW, Altig R (1999). Body plan: development and morphology. Tadpoles: The Biology of Anuran Larvae.

[ref-3] Bokermann WCA (1966). Lista Anotada das Localidades Tipo de Anfíbios Brasileiros.

[ref-4] Borges SH, Cornelius C, Ribas CC, Almeida R, Guilherme E, Aleixo A, Dantas S, Santos MPD, Moreira M (2016). What is the avifauna of Amazonian white-sand vegetation?. Bird Conservation International.

[ref-5] Caldwell JP, Lima AP, Keller C (2002). Redescription of *Colostethus marchesianus* (Melin, 1941) from its type locality. Copeia.

[ref-6] Cochran DM, Goin CJ (1970). Frogs of Colombia. Bulletin of the United States National Museum.

[ref-8] De Carvalho VT, De Fraga R, Bittencourt S, Bonora L, Condrati LH, Gordo M, Vogt RC (2018). Geographic distribution of *Aparasphenodon venezolanus* (Anura: Hylidae) in the Brazilian Amazon lowlands. Phyllomedusa.

[ref-9] Drummond AJ, Suchard MA, Xie D, Rambaut A (2012). Bayesian phylogenetics with BEAUti and the BEAST 1.7. Molecular Biology and Evolution.

[ref-10] Duellman WE (1970). The hylid frogs of middle America/William E. Duellman.

[ref-13] Faivovich J, García PCA, Ananias F, Lanari L, Basso NG, Wheeler WC (2004). A molecular perspective on the phylogeny of the *Hyla pulchella* species group (Anura, Hylidae). Molecular Phylogenetics and Evolution.

[ref-14] Faivovich J, Haddad CFB, Garcia PCA, Frost DR, Campbell JA, Wheeler WC (2005). Systematic review of the frog family Hylidae, with special reference to Hylinae: phylogenetic analysis and taxonomic revision. Bulletin of the American Museum of Natural History.

[ref-15] Fouquet A, Gilles A, Vences M, Marty C, Blanc M, Gemmell NJ (2007). Underestimation of species richness in neotropical frogs revealed by mtDNA analyses. PLOS ONE.

[ref-16] Frost DR (2019). Amphibian species of the world: an online reference. http://research.amnh.org/herpetology/amphibia/index.html.

[ref-17] Gordo M, Neckel-Oliveira S (2004). Osteocephalus planiceps. Herpetological Review.

[ref-18] Gosner KL (1960). A simplified table for staging anuran embryos and larvae with notes on identification. Herpetologica.

[ref-19] Guindon S, Dufayard JF, Lefort V, Anisimova M, Hordijk W, Gascuel O (2010). New algorithms and methods to estimate maximum-likelihood phylogenies: assessing the performance of PhyML 3.0. Systematic Biology.

[ref-20] Hall TA (1999). BioEdit: a user-friendly biological sequence alignment editor and analysis program for Windows 95/98/NT. Nucleic Acids Symposium Series.

[ref-21] Heyer WR, Rand AS, Cruz CAG, Peixoto OL, Nelson CE (1990). Frogs of Boracéia. Arquivos de Zoologia.

[ref-22] Hoogmoed MS (2013). Rediscovery of the rare tree frog *Hyla inframaculata* Boulenger, 1882 (Anura: Hylidae), in Amazonian Brazil with notes on variation and distribution, and its generic allocation. Amphibia-Reptilia.

[ref-23] Instituto Brasileiro de Geografia e Estatística (1992). Instituto Brasileiro de Geografia e Estatística, Recursos naturais e meio ambiente: uma visão do Brasil.

[ref-24] Jowers MJ, Downie JR, Cohen BL (2008). The golden tree frog of Trinidad, *Phyllodytes auratus* (Anura: Hylidae): systematic and conservation status. Studies on Neotropical Fauna and Environment.

[ref-25] Jungfer K-H (2010). The taxonomic status of some spiny-backed treefrogs, genus *Osteocephalus* (Amphibia: Anura: Hylidae). Zootaxa.

[ref-27] Jungfer K-H, Faivovich J, Padial JM, Castroviejo-Fisher S, Lyra MM, Berneck BVM, Iglesias PP, Kok PJR, MacCulloch RD, Rodrigues MT, Verdade VK, Torres Gastello CP, Chaparro JC, Valdujo PH, Reichle S, Moravec J, Gvoždík V, Gagliardi-Urrutia G, Ernst R, De la Riva I, Means DB, Lima AP, Señaris JC, Wheeler WC, Haddad CFB (2013). Systematics of spiny-backed treefrogs (Hylidae: *Osteocephalus*): an Amazonian puzzle. Zoologica Scripta.

[ref-28] Jungfer K-H, Hödl W (2002). A new species of *Osteocephalus* from Ecuador and a redescription of *O. leprieurii* (Duméril & Bibron, 1841) (Anura: Hylidae). Amphibia-Reptilia.

[ref-29] Jungfer K-H, Lehr E (2001). A new species of *Osteocephalus* with bicoloured iris from Pozuzo (Peru: Departamento de Pasco) (Amphibia: Anura: Hylidae). Zoologische Abhandlungen Staatliches Museum für Tierkunde Dresden.

[ref-30] Jungfer K-H, Ron S, Seipp R, Almendáriz A (2000). Two new species of hylid frogs, genus *Osteocephalus*, from Amazonian Ecuador. Amphibia-Reptilia.

[ref-31] Jungfer K-H, Schiesari LC (1995). Description of a central Amazonian and Guianan tree frog, genus *Osteocephalus* (Anura, Hylidae), with oophagous tadpoles. Alytes.

[ref-32] Jungfer K-H, Verdade VK, Faivovich J, Rodrigues MT (2016). A new species of spiny-backed treefrog (*Osteocephalus*) from Central Amazonian Brazil (Amphibia: Anura: Hylidae). Zootaxa.

[ref-33] Kearse M, Moir R, Wilson A, Stones-Havas S, Cheung M, Sturrock S, Buxton S, Cooper A, Markowitz S, Duran C, Thierer T, Ashton B, Meintjes P, Drummond A (2012). Geneious Basic: an integrated and extendable desktop software platform for the organization and analysis of sequence data. Bioinformatics.

[ref-34] Kimura M (1980). A simple method for estimating evolutionary rates of base substitutions through comparative studies of nucleotide sequences. Journal of Molecular Evolution.

[ref-35] Köhler J, Jansen M, Rodriguez A, Kok PJR, Toledo LF, Emmrich M, Glaw F, Haddad CFB, Rödel MO, Vences M (2017). The use of bioacoustics in anuran taxonomy: theory, terminology, methods and recommendations for best practice. Zootaxa.

[ref-36] Lampo M, Chacón AF, Nava F, Molina C, Señaris JC, Lampo M, Rial A (2009). Acervo genético. Anfibios De Venezuela: Estado Del Conocimiento Y Reccomendaciones Para Su Conservación.

[ref-37] Lanfear R, Frandsen PB, Wright AM, Senfeld T, Calcott B (2017). PartitionFinder 2: new methods for selecting partitioned models of evolution for molecular and morphological phylogenetic analyses. Molecular Biology and Evolution.

[ref-38] Lima AP, Magnusson WE, Menin M, Erdtmann LK, Rodrigues DJ, Keller C, Hödl W (2006). Guia de sapos da Reserva Adolpho Ducke, Amazônia Central: Guide to the Frogs of Reserva Adolpho Ducke, Central Amazonia.

[ref-40] Maddison WP, Maddison DR (2018). https://www.mesquiteproject.org/.

[ref-42] Melin DE (1941). Contributions to the knowledge of the Amphibia of South America: Göteborgs Kungl. Vetenskaps-och Vitterhets-samhälles. Handlingar. Serien B, Matematiska och Naturvetenskapliga Skrifter.

[ref-43] Menin M, De Carvalho VT, Almeida AP, Gordo M, Oliveira DP, Luiz LF, Campos JV, Hrbek T (2017). Amphibians from Santa Isabel do Rio Negro, Brazilian Amazonia. Phyllomedusa: Journal of Herpetology.

[ref-44] Moravec J, Aparicio J, Guerrero-Reinhard M, Calderon G, Jungfer K-H, Gvoždík V (2009). A new species of *Osteocephalus* (Anura: Hylidae) from Amazonian Bolivia: first evidence of tree frog breeding in fruit capsules of the Brazil nut tree. Zootaxa.

[ref-45] Moravec J, Tuanama IA, Gagliardi-Urrutia G, Gvoždík V (2016). Amphibians and reptiles recorded in the conservation area Imiría in the Ucayali region in Peru. Acta Societatis Zoologicae Bohemicae.

[ref-46] Myers CW, Duellman WE (1982). A new species of *Hyla* from Cerro Colorado, and other tree frog records and geographical notes from western Panama. American Museum Novitates.

[ref-47] Neckel-Oliveira S, Gordo M, Borges SH, Iwanaga S, Durigan CC, Pinheiro MC (2004). Anfíbios, lagartos e serpentes do Parque Nacional do Jaú. Janelas Para A Biodiversidade No Parque Nacional Do Jaú: Uma Estratégia Para O Estudo Da Biodiversidade Na Amazônia.

[ref-49] Palumbi SR, Martin AP, Romano SL, McMillan WO, Stice L, Grabowski G (1991). The sample tool’s guide to PCR.

[ref-50] R Core Team (2018). A language and environment for statistical computing.

[ref-51] Rambaut A, Drummond AJ, Xie D, Baele G, Suchard MA (2018). Posterior summarisation in Bayesian phylogenetics using Tracer 1.7. Systematic Biology.

[ref-52] Randrianiaina R-D, Strauß A, Glos J, Glaw F, Vences M, Arntzen JW (2011). Diversity, external morphology and ‘reverse taxonomy’ in the specialized tadpoles of Malagasy river bank frogs of the subgenus *Ochthomantis* (genus *Mantidactylus*). Contributions to Zoology.

[ref-53] Ron SR, Merino-Viteri A, Ortiz DA (2019). https://bioweb.bio/faunaweb/amphibiaweb.

[ref-54] Ron S, Pramuk JB (1999). A new species of *Osteocephalus* (Anura: Hylidae) from Amazonian Ecuador and Peru. Herpetologica.

[ref-55] Ron S, Toral E, Venegas PJ, Barnes CW (2010). Taxonomic revision and phylogenetic position of *Osteocephalus festae* (Anura, Hylidae) with description of its larva. ZooKeys.

[ref-56] Ron SR, Venegas PJ, Toral E, Read M, Ortiz DA, Manzano AL (2012). Systematics of the *Osteocephalus buckleyi* species complex (Anura, Hylidae) from Ecuador and Peru. ZooKeys.

[ref-57] Ronquist F, Teslenko M, Van Der Mark P, Ayres DL, Darling A, Höhna S, Larget B, Liu L, Suchard MA, Huelsenbeck JP (2012). MrBayes 3.2: efficient Bayesian phylogenetic inference and model choice across a large model space. Systematic Biology.

[ref-58] Salerno PE, Ron SR, Señaris JC, Rojas-Runjaic FJM, Noonan BP, Cannatella DC (2012). Ancient Tepui summits harbor young rather than old lineages of endemic frogs. Evolution.

[ref-59] Savage JM, Heyer WR (1967). Variation and distribution in the tree-frog genus *Phyllomedusa* in Costa Rica, Central America. Beiträge zur Neotropischen Fauna.

[ref-60] Schulze A, Jansen M, Köhler G (2015). Tadpole diversity of Bolivia’s lowland anuran communities: molecular identification, morphological characterization, and ecological assignment. Zootaxa.

[ref-61] Sueur J, Aubin T, Simonis C (2008). Seewave, a free modular tool for sound analysis and synthesis. Bioacoustics: International Journal of Animal Sound and its Recording.

[ref-62] Tamura K, Stecher G, Peterson D, Filipski A, Kumar S (2013). MEGA6: molecular evolutionary genetics analysis version 6.0. Molecular Biology and Evolution.

[ref-63] Thompson JD, Higgins DG, Gibson TJ (1994). Clustal W: improving the sensitivity of progressive multiple sequence alignment through sequence weighting, position-specific gap penalties and weight matrix choice. Nucleic Acids Research.

[ref-64] Trueb L, Duellman WE (1971). A synopsis of neotropical hylid frogs, genus *Osteocephalus*. Occasional Papers of the Museum of Natural History, The University of Kansas.

[ref-65] Wickham H (2016). ggplot2: elegant graphics for data analysis.

[ref-66] Wiens JJ, Graham CH, Moen DS, Smith SA, Reeder TW (2006). Evolutionary and ecological causes of the latitudinal diversity gradient in hylid frogs: treefrog trees unearth the roots of high tropical diversity. American Naturalist.

[ref-67] Wiens JJ, Kuczynski CA, Hua X, Moen DS (2010). An expanded phylogeny of treefrogs (Hylidae) based on nuclear and mitochondrial sequence data. Molecular Phylogenetics and Evolution.

